# Impact of smartphone-assisted prenatal home visits on women’s use of facility delivery: Results from a cluster-randomized trial in rural Tanzania

**DOI:** 10.1371/journal.pone.0199400

**Published:** 2018-06-18

**Authors:** Kristy Hackett, Curtis Lafleur, Peter Nyella, Ophira Ginsburg, Wendy Lou, Daniel Sellen

**Affiliations:** 1 Department of Global Health and Population, Harvard T.H. Chan School of Public Health, Harvard University, Boston, Massachusetts, United States of America; 2 Dalla Lana School of Public Health, University of Toronto, Toronto, Ontario, Canada; 3 Ontario Shores Centre for Mental Health Sciences, Whitby, Ontario, Canada; 4 Irish Aid Tanzania, Dar es Salaam, Tanzania; 5 Section for Global Health, Department of Population Health, New York University Langone Health, New York, New York, United States of America; 6 Department of Anthropology, University of Toronto, Toronto, Ontario, Canada; 7 Department of Nutritional Sciences, University of Toronto, Ontario, Canada; TNO, NETHERLANDS

## Abstract

**Background:**

About half of births in rural Tanzania are assisted by skilled providers. Point-of-care mobile phone applications hold promise in boosting job support for community health workers aiming to ensure safe motherhood through increased facility delivery awareness, access and uptake. We conducted a controlled comparison to evaluate a smartphone-based application designed to assist community health workers with data collection, education delivery, gestational danger sign identification, and referrals.

**Methods:**

Community health workers in 32 randomly selected villages were cluster-randomized to training on either smartphone (intervention) or paper-based (control) protocols for use during household visits with pregnant women. The primary outcome measure was postnatal report of delivery location by 572 women randomly selected to participate in a survey conducted by home visit. A mixed-effects model was used to account for clustering of subjects and other measured factors influencing facility delivery.

**Findings:**

The smartphone intervention was associated with significantly higher facility delivery: 74% of mothers in intervention areas delivered at or in transit to a health facility, versus 63% in control areas. The odds of facility delivery among women counseled by smartphone-assisted health workers were double the odds among women living in control villages (OR, 1.96; CI, 1.21–3.19; adjusted analyses). Women in intervention areas were more likely to receive two or more visits from a community health worker during pregnancy than women in the control group (72% vs. 60%; chi-square = 6.9; p < 0.01). Previous facility delivery, uptake of antenatal care, and distance to the nearest facility were also strong independent predictors of facility delivery.

**Interpretation:**

Community health worker use of smartphones increased facility delivery, likely through increased frequency of prenatal home visits. Smartphone-based job aids may enhance community health worker support and effectiveness as one component of intervention packages targeting safe motherhood.

**Trial registration:**

NCT03161184.

## Introduction

Since the advent of the Safe Motherhood Initiative nearly thirty years ago [1], leaders in maternal and neonatal health have called for improved access to skilled medical professionals during labour and delivery. This advocacy reflects evidence that timely provision of quality facility-based delivery services by trained providers remains the best strategy available for reducing maternal deaths, a majority of which occur during the intrapartum period [2]. Studies show that facility delivery improves maternal survival rates in low and middle-income countries (LMICs) because trained medical personnel can react promptly to sudden obstetric complications [3–5].

Despite global recognition of these benefits, ensuring universal access to safe facility delivery services remains a significant challenge in many LMICs, and particularly in rural areas of south Asia and sub-Saharan Africa [6]. Documented “supply-side” challenges include the scarcity and unequal distribution of properly equipped health facilities and human resources for health, and “demand-side” factors such as preference for home birth and/or village-based traditional birth attendants, community concerns about facility delivery and service quality, low awareness and recognition of pregnancy and obstetric danger signs and gendered inequities in household decision making [7–12]. To improve facility delivery rates in low-resource settings, existing human resources for health must be leveraged to strengthen links between households, communities and the formal healthcare system.

Frontline community health workers (CHW), a cadre of peer-elected volunteers trained to deliver basic medical and health promotion services, can be powerful drivers of maternal health service demand, particularly in rural populations. While they cannot replace highly skilled healthcare professionals, motivated and well supervised CHW equipped with appropriate knowledge, experience and skills can help to mobilize communities by delivering preventive health education and services and promoting health-seeking behaviors [13]. Specifically, CHW can help to improve facility delivery coverage through working with families to plan for safe delivery, identify danger signs during pregnancy, and make timely referrals to skilled clinicians during pregnancy and childbirth. Despite this potential, CHW performance is limited in many settings by the absence of sufficient training, ongoing supportive supervision and job aids [13, 14]. Capacity and performance gaps linked to equity of service delivery, training and supervision, incentives, attrition, record keeping and information management are subjects of ongoing debate among programmers and policy makers globally [14, 15].

To improve the quality of CHW services, innovative strategies are needed to better support their work. The field of mobile health (‘mHealth’), which involves the use of mobile technologies such as cell phones to improve the delivery of, and access to, health services and information [16], offers potential solutions. For example, mHealth leverages wireless mobile technologies to support delivery and management of existing evidence-based health interventions [17], including those delivered by CHW. Various countries have implemented programs that use mobile phone-based approaches to support data collection, emergency medical response, point-of-care diagnostics and support for health workers, and health promotion campaigns [18]. Proponents of mHealth strategies suggest that such simple, low-cost mobile interventions could generate significant health gains in sub-Saharan Africa, particularly among women and children under five [19].

While mHealth interventions hold promise, rigorous scientific studies evaluating their impact remain scarce [17]. Most research on mobile phones as a tool for maternal health behaviour change has been conducted in high-income countries [20]. Among the few studies from LMICs, the vast majority report on the effects of health-related SMS (text) messaging on patient health behaviours [21, 22] and to a lesser extent, direct voice support for patients [23, 24], or a combination of both [25]. While a number of studies on usability, feasibility and acceptability of mHealth strategies report positive findings [22, 26, 27] research evaluating the health impacts of cell phone-based strategies for frontline health workers has been mostly descriptive and remains inconclusive [17].

Reviews have called for a more robust evidence base for mHealth approaches to inform the design and implementation of MNCH programs, and broader funding and policy-related decision making [28]. At the time of writing, only one published study reports on the impact of mHealth initiatives on skilled birth attendance [29]; and only one (a quasi-experimental study in India) has assessed whether point-of-care support systems for frontline health workers can improve facility delivery [30]. We aim to narrow this evidence gap by reporting on the impact of a smartphone-based application for CHW on women’s utilization of facility delivery in rural Tanzania.

### Programmatic context and study setting

This study evaluated an intervention aimed at increasing women’s demand for, and utilization of, facility delivery within the context of a large maternal, newborn and child health (MNCH) project in Tanzania, where maternal mortality remains relatively high (432/100,000 live births) [31] and only 63% of deliveries are conducted in health facilities [32]. Striking wealth disparities exist across Tanzania: among women in the highest wealth quintile, 95% deliver with assistance from a skilled provider, versus only 42% within the lowest quintile [32]. Coverage also varies widely between regions, with estimates ranging between 40% and 90% across the country (32). Women with lower education, higher parity, and those living in rural areas are the least likely to access facility delivery services [9, 32, 33].

Supporting Systems to Improve Nutrition, Maternal, Newborn, and Child Health (SUSTAIN-MNCH) was a three-year project implemented by World Vision through various governmental and non-governmental partners in two districts of the country’s central Singida Region. Singida is one of Tanzania’s poorest regions, home to about 1.4 million people, 60% of whom fall within the lowest two national wealth quintiles [32]. The region is characterized by a weak health infrastructure (only 158 health facilities as of 2009 –the second lowest per capita in the country) [34] and a high maternal mortality ratio (468 per 100,000 live births) [31]. While almost all women in Singida access antenatal care (ANC) at least once during pregnancy, only 60% attend 4 or more ANC visits, and approximately 40% deliver without assistance from a skilled birth attendant [32]. Among many activities targeting improved MNCH outcomes, the SUSTAIN project aimed to strengthen CHW capacity through roll-out of a smartphone-based job aid application designed to facilitate household visits, counseling, and collection of information on key MNCH indicators.

### Tanzania iMNCH program background

A fragmented health infrastructure and predominantly rural population (approximately 75% of inhabitants) mean Tanzanian CHW provide a crucial link between hard-to-reach communities and the formal health system [35]. At the time of the study, national recommendations were for each village health committee to appoint a minimum of two CHW, ideally one male and one female. These individuals are selected by their community, work on a voluntary basis and must have at least “form 4” (primary) level education. Most villages in target areas had existing CHW in place when the SUSTAIN project began, but levels of activity, experience, training and expectations varied widely.

In 2012 the Tanzania Ministry of Health & Social Welfare approved an integrated Maternal Newborn and Child Health (iMNCH) training program for CHW [36] to standardize CHW duties and expectations across the country. This three-week curriculum trains CHW to make regular household visits at specific times throughout pregnancy, infancy and early childhood to educate families on birth preparedness, infant feeding and nutrition, and the importance of ANC attendance and facility delivery, among other topics. CHW learn to identify danger signs during pregnancy and the postpartum period, and to refer clients to health facilities accordingly. CHW are provided with paper-based registers, and photo books (*bango kititas* in Kiswahili), which are used as counselling tools during household visits to promote healthy behaviours. All CHW in SUSTAIN project areas were trained on the national iMNCH program prior to implementing the smartphone intervention.

### The SUSTAIN smartphone application

Aiming to strengthen support for CHW in SUSTAIN target areas, World Vision collaborated with D-Tree International to develop a smartphone application for use by CHW during household visits. The application was developed using CommCare [37], an open source platform designed specifically for use by frontline health workers.

The SUSTAIN application was developed in accordance with national iMNCH program guidelines and was intended for use along with the iMNCH photo books during household counseling sessions with clients. Basic functions of the application include client registration, home visit scheduling, time-tailored counseling prompts, automated referral and follow-up reminders, and data management and reporting support. The application is used by CHW to register pregnant women as soon as they are identified at the village level. Once registered, the tool prompts CHW to monitor the status of clients throughout pregnancy and following delivery.

During prenatal household visits, the application guides CHW through electronic “decision tree” protocols, directing them to specific health counselling topics and messages based on the woman’s gestational age, and her answers to various diagnostic questions. The tool directs CHW to particular lessons in the photo book and reminds them to counsel women on the benefits of seeking antenatal care, developing a birth plan, and seeking skilled birth assistance at health facilities. The application also assists with danger sign identification, flags clients who require immediate referral to health facilities, and reminds CHW to follow up with clients previously referred.

We hypothesized that use of this smartphone application as a behaviour change communication tool during prenatal visits would lead to increased utilization of facility delivery among CHW clients through improved quality of targeted counseling, provision of more standardized care, and an improved referral and follow-up system.

## Methods

### Ethical considerations

The Tanzanian National Institute for Medical Research (NIMR), the Tanzanian Commission for Science and Technology (COSTECH) and the Office of Research Ethics at the University of Toronto, Canada approved the research protocol, and COSTECH issued a research permit to the first author to conduct primary data collection in Singida Region. Written permission to conduct the study was obtained from regional, and district-level authorities prior to the start of data collection. Mothers aged 16–49 were eligible to participate in this study; we did not obtain parental consent for minors under 18 years of age. Both IRBs (University of Toronto and NIMR in Tanzania) approved this consent protocol (Reference # 28832, S1 Protocol).

The study was not registered prior to participant enrollment because the institutional Research Ethics Board (REB) advised authors at the outset that this study did not fit criteria for clinical trial registration. This is stated in a letter of support from the Director of the University of Toronto REB (S1 Supporting Information). At the time of submission, PLOS One editors requested retrospective trial registration (NCT03161184). The authors confirm that there are no other ongoing or related trials for this intervention.

### Evaluation study design

We used a cluster-randomized, controlled comparison design to evaluate the impact of implementation of the SUSTAIN smartphone application on facility delivery from August 2013 to June 2014. The intervention was implemented in two World Vision longer term area development programmes in Singida Rural and Iramba districts where the SUSTAIN project was also being implemented.

Following national iMNCH guidelines, World Vision trained two CHW in each village, typically one male and one female. CHW pairs from intervention villages were trained to make household visits using both the iMNCH photo book and the smartphone application (combined intervention referred to as “SP+”). CHW from control villages were trained only on the iMNCH photo book and standard paper-based protocols. CHW in both study groups followed their respective protocols for a period of approximately 10 months before postnatal outcome measures were assessed.

### Intervention allocation

**Fig 1:**
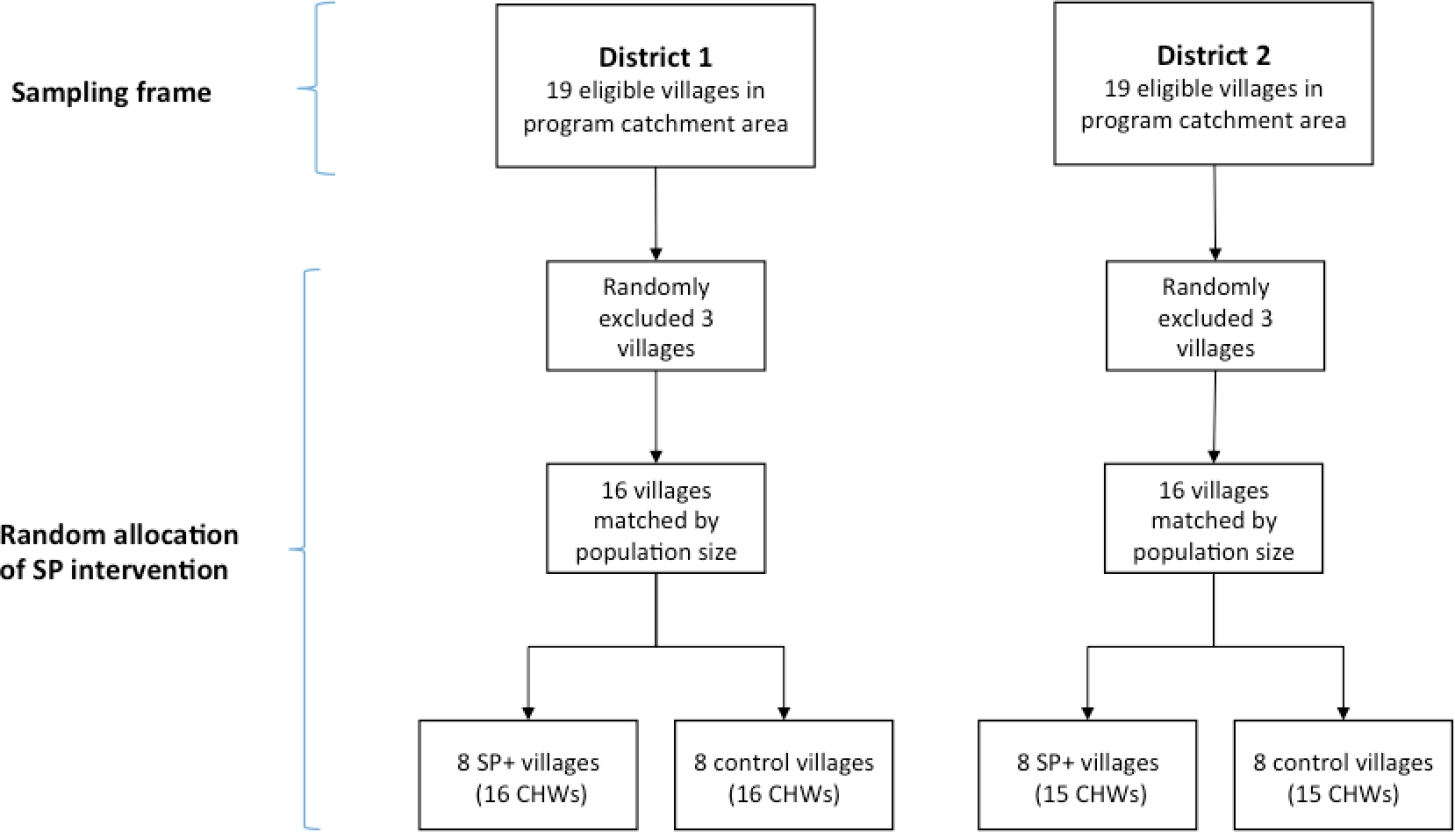
Study design overview (attached as separate file).

At the time of the study, World Vision’s longer term area development programme covered 19 villages in each district, therefore our sampling frame consisted of 38 villages in total (Fig 1). After stratifying by district and pair-matching villages based on population size, we randomly selected villages to be either SP+ or control clusters. Thirty-two smartphones set up with the application were randomly allocated to 16 pairs of CHW. An additional 16 villages (and thus 16 CHW pairs) were randomly assigned to the control group (total N = 64). Group assignment was unmasked. In each district, we selected intervention villages using a 3-step protocol: (i) a simple randomization procedure was used to select 3 (out of a possible 19) villages to exclude from the study; (ii) the remaining 16 villages were pair-matched on population size; (iii) for each matched pair, one was randomly allocated to the intervention arm and the other to the control arm.

### Study population

We recruited CHW into the study during iMNCH training workshops at baseline. Eligible CHW were actively working in one of the two study districts and had completed the national iMNCH training program. At the end of the study period we recruited women who had recently delivered and were clients of eligible CHW during their most recent pregnancy. Eligible women clients were: a) between the ages of 16–49 years; b) pregnant during the intervention period (a prerequisite for exposure to prenatal counselling by CHW); and c) had a live birth and the child was still living. The last criterion was a deviation from the originally approved protocol. This change was made at the time of data collection based on a rationale that it would be unethical to interview women who had recently lost a child regarding their childbirth experiences. When these cases were encountered during data collection (N = 8), field staff reported them to the village CHW and to World Vision, who followed established protocols for district health system reporting.

Enrollment of CHWs occurred on July 23^rd^, 2013. Enrollment of women clients occurred over a period of approximately six weeks in May and June, 2014.

### Sample size calculation for household surveys

The required sample size for household surveys was estimated using methods outlined by Campbell et al (2004) [38]. As these authors outline, accounting for the effect of clustering on sample size requirements can be achieved statistically using an appropriate intra-cluster correlation coefficient (ICC) value, which is based on the ratio of the *between cluster* to *within-cluster* variance for a given outcome of interest. To identify a suitable ICC, we reviewed the literature on cluster RCTs that assessed skilled birth attendance as a main outcome. We determined that an appropriate ICC estimate was 0.14 (the average ICC value from two studies conducted in neighboring Malawi described by Pagel and colleagues) [39, 40]. We calculated a total sample size of 596 women (298 in each study arm) would allow for 80% power to detect in either direction at least a 12% difference between the experimental and control groups, at a two-sided significance level of 5%, using the method of Campbell et al. [38], and assuming 32 villages (clusters) equally distributed between study arms. Using the equation outlined by Campbell et al. and assuming 32 villages (clusters), we calculated a total sample size of 596 women (298 in each study arm). This sample size allowed for detection of a 12% difference between the intervention and control groups, at a 5% significance level (two-sided) and 80% power. We anticipated a 30% loss to follow-up (due to refusal, travel, illness or other causes) and oversampled accordingly. Based on these calculations, we aimed to recruit at least 24 women from each village.

### Sampling & recruitment

CHW from selected villages (N = 64) were recruited through the iMNCH training program that World Vision facilitated in SUSTAIN project areas, and all were invited to participate at the end of the three-week training period. All invited CHW agreed to participate, provided written informed consent and completed a baseline survey upon enrollment.

Women participants were recruited for postnatal household surveys at the end of the intervention period, using the following sampling protocol: (i) we obtained from each CHW complete lists of clients meeting study inclusion criteria; (ii) if a CHW had more than 12 eligible women on their list, we prioritized those with longer exposure time (i.e. visited by a CHW prior to 6 months gestation); (iii) we randomly selected from remaining client names until N = 12 for each CHW; (iv) if a CHW had less than 12 eligible clients, we invited all to participate. In these cases, we selected additional clients from the second CHW’s list to make up the difference; (v) in villages where CHW had more than 24 eligible clients, we randomly selected up to 24 names; (vi) if a selected woman was not present (e.g. travelling, relocated, or unreachable) or found to be ineligible on the day of the survey, we replaced this client with another randomly selected name, if possible. All women provided written informed consent prior to beginning the survey. The trial ended once the sample size goal was reached.

### Outcome measures

The main outcome measured was delivery at, or while in transit to, a health facility among a cluster-randomized sample of women who delivered between 3 and 9 months post-baseline (defined as the time of deployment of trained CHW on iMNCH or SP+). The rationale for grouping facility deliveries with deliveries ‘in transit’ is as follows: Monthly household visits by CHW are intended to increase women’s knowledge and awareness of the importance of delivering at a facility. Since this was a behaviour change intervention, we reasoned that women who delivered on the way to a facility had the *intention* to deliver at a facility, and thus we grouped them together in the primary analysis. This is analogous to the concept of “intention-to-treat” analyses of clinical trials.

To ascertain the main outcome we conducted postnatal household surveys with a randomly selected sample of eligible mothers approximately 10 months following baseline training. Exposure time (to either SP+ or paper-based protocols) was the same for both study groups. Women were asked whether they delivered at home, a dispensary, a health centre, a hospital, or in transit to a health facility. Interviewers confirmed this information by checking participants’ clinic cards whenever possible. Research team members used tablets to administer surveys in private and without CHW or NGO staff present. A complete list of secondary outcome measures (results not reported in this manuscript) is available (S1 Table).

### Loss to follow-up

Two CHW from different villages (both intervention villages in Iramba district) dropped out of the study shortly after the iMNCH training seminar due to conflicting household responsibilities and were not actively engaged with CHW duties throughout the study period. In these cases, which resulted in only a single CHW active in each of the two clusters, we sampled only from the remaining CHW’s client lists for household surveys. Furthermore, a pair of CHW from the same cluster control village did not perform any assigned tasks following training, expressing concerns about lack of compensation for their work. We excluded this village from the household survey because women were not exposed to either of the two interventions and were therefore ineligible post-randomization. We replaced this cluster-control group by randomly selecting one of the previously excluded villages for inclusion in the household survey. This was a conservative approach to estimating the impact of the SP intervention since including a control village with *no* CHW activity was likely to result in underestimating the relative influence of paper-based iMNCH protocols.

### Measurement of potential confounding factors

Proxy measures of socioeconomic status included water source, and toilet type, but not household income because we anticipated that asking women directly about income would yield uncertain precision and validity. To account for the potential confounding effect of health facility quality and service readiness, we developed multivariate measures, derived from previously validated tools [41, 42], to assess two dimensions: 1) perceived quality of the nearest facility according to women, captured by Likert-scale questionnaires; and b) MNCH service readiness of health facilities measured using a 47-item health facility checklist. All study tools were piloted with a small sample of women (N = 10) who were external to the study but lived within program areas and therefore had similar exposure to health services and similar socioeconomic profiles as study participants. Where appropriate, tools were modified to reflect local language, literacy levels and cultural interpretations prior to use.

### Statistical Analysis

We assessed the effect of smartphone-supported counseling on the primary outcome by comparing differences in the likelihood of home versus delivery at or in transit to a health facility in each study arm approximately 10 months post-intervention. Statistical analyses were completed using SPSS for MAC software, version 22.0. We used the variance components procedure for mixed-effects models to account for clustering of subjects at three levels: within districts, within pair-matched villages, and within individual villages. The significance level was set at 5% for all two-side tests. All analyses were performed by intention to treat.

We fitted an initial model including all variables believed to influence women’s delivery location (Table 1), including two-way interactions. Models were built using an iterative backwards step-wise progression as follows: (i) all individual-level factors (e.g. age, parity, religion) were added to the model; (2) we ran the model then removed all non-significant variables (p > 0.05); (iii) we added all community-level factors (e.g. district, distance from HF), ran the model and removed all non-significant variables; (iv) finally, we added in all health systems related variables (e.g. quality of HF/CHW scores) then removed insignificant variables to arrive at the final reduced model. For all models, results were expressed as odds ratios (ORs) for facility delivery with 95% confidence intervals and significance was defined as p < 0.05.

**Table 1:** Variables included in the initial model.

Individual-level factors	Community-level factors	Health system-level factors
Maternal age, parity, religion, socioeconomic status (toilet source, water source), previous facility delivery (at least one), history of birth complications, timing of first ANC visit, number of ANC visits.	District, distance to the nearest health facility, distance to the nearest hospital.	Quality of the nearest health facility (checklist score), perceived quality of CHW care (score), number of prenatal visits by a CHW

## Results

### Achieved sample size

**Fig 2:**
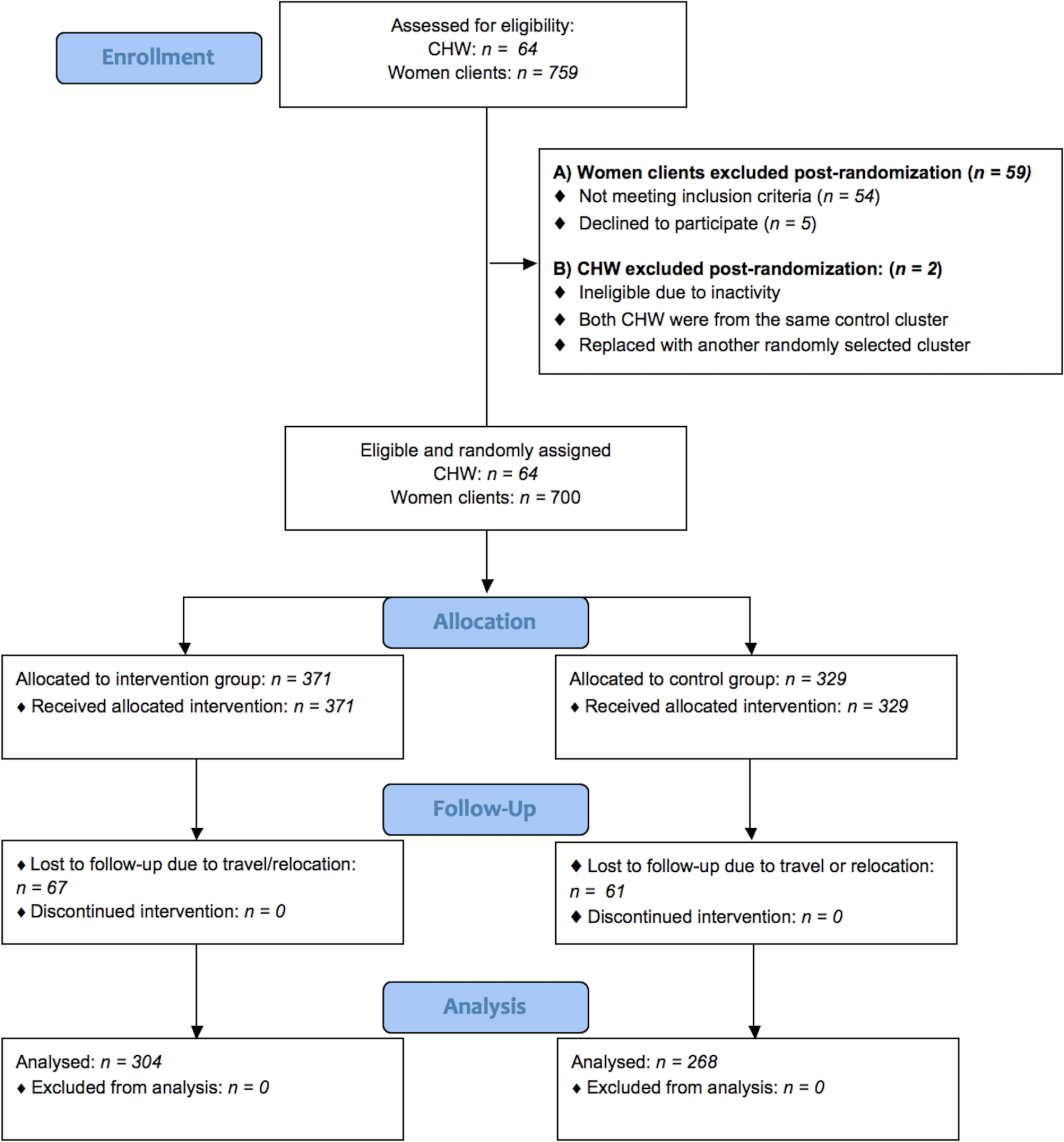
Study flow diagram outlining sample size achieved in each study arm.

A total of 572 postnatal household surveys were conducted with women clients. The numbers of women clients randomized and included in the primary analysis are outlined in Fig 2. A team of ten interviewers conducted surveys over a three-week period in June 2014.

### Descriptive characteristics

Sociodemographic profiles of women in SP+ and control groups are presented in Tables 2 and 3. Significance was assessed using Independent samples t-tests or Pearson’s chi-squared tests. A majority of women in both groups had primary school education. Of those who specified a religion, the proportions identifying as Christian and Muslim (the two predominant religions in Tanzania) were not significantly different between intervention and control groups. Most women lived within the median distance (3.3 km) from any health facility (usually a lower level dispensary), but the majority lived further than the median distance (10.2 km) from the nearest hospital. While median distances to the nearest health facility did not differ between study groups, a significantly higher proportion of women in the control group (77%) lived close (defined as < 5km to the nearest health facility), compared to 67% of women in the SP+ group (p = 0.02, assessed by Pearson’s chi-square test).

**Table 2:** Sociodemographic profiles of women participating in household surveys.

Independent variables	SP+ (N = 304)		Control (N = 268)		p-value*
Maternal age, y (*mean*, *standard error)*	28.2	0.4	29.1	0.4	0.12
Infant age, mo (*mean*, *standard error)*	3.2	0.1	3.0	0.1	0.29
Religion** (N, %)					
Christian	194	64.0	157	61.6	0.56
Muslim	109	36.0	98	38.4	
Highest level of schooling (N, %)					
None	39	12.8	50	18.7	0.15
Primary	252	82.9	102	75.4	
Secondary	13	4.3	16	6.0	
Water source (N, %)					
Access to closed/protected source	134	44.1	98	36.6	0.07
Unprotected/open water source	170	55.9	170	63.4	
Type of toilet facilities, (N, %)					
VIP latrine or flush toilet	0	0.0	1	0.4	0.35
Traditional/open pit latrine	299	98.4	265	98.9	
No facilities, bush or field	5	1.6	2	0.7	
Distance from the nearest health facility, km					
Mean, *standard error*	3.8	0.1	3.4	0.1	0.09
Median, *standard deviation*	3.5	2.4	3.2	2.1	0.32
Distance from nearest hospital, km					
Mean, *standard error*	11.3	0.4	12.1	0.5	0.14
Median, *standard deviation*	9.1	6.4	10.7	7.5	0.03

*Significance assessed by Independent samples t-tests, or Pearson’s chi-squared tests

**Of those women who specified a religion (N = 558)

**Table 3:** Obstetric risk indicators of women participating in household surveys.

Independent variables	SP+ (N = 304)		Control (N = 268)		p-value*
Parity (*mean*, *standard error)*	3.6	0.4	3.9	0.1	0.04
Parity prior to most recent birth (N, %)					
Nullipara (0 previous births)	62	20.4	47	17.5	0.40
Multipara (1 or more births)	242	79.6	221	82.5	
Previously delivered at least one child in a HF	214	70.4	193	72.0	0.78
History of obstetric complication(s)	46	15.1	37	13.8	0.64
ANC visits for most recent child (N, %)					
<4	180	59.2	157	58.6	0.93
≥4	124	40.8	111	41.4	
Gestational age at first ANC visit (N, %)					
<6 months	143	47.4	136	50.7	0.45
≥6 months	159	52.6	132	49.3	

*Significance assessed by Independent samples t-tests, or Pearson’s chi-squared tests

### Impact of SP+ on facility-based delivery

Overall, SP+ was associated with a significantly higher rate of facility delivery: 74% of mothers in the intervention group delivered at or in transit to a health facility, versus 62% in the control group (Table 4). Among women who delivered at a facility, 71% did so at a hospital; of these women, 65% had bypassed a nearer health facility (dispensary or health centre) to deliver at a hospital.

**Table 4:** Location of birth following exposure to each intervention.

	Total (N = 572)		SP+ (N = 304)		Control (N = 268)	
Reported Delivery Location* ***(%*, *N)***						
Home	31.6	181	26.3	80	37.7	101
Dispensary	10.5	60	10.5	32	10.4	28
Health Centre	8.0	46	9.2	28	6.7	18
Hospital	44.6	255	47.0	143	41.8	112
In transit	5.2	30	6.9	21	3.4	9

* By retrospective report at interview, after delivery; confirmed by crosschecking clinic cards when available.

In Iramba district, 89.6% of women delivered at or on the way to a facility, compared to only 48.3% in Singida Rural district. After accounting for the effects of district and village-level clustering and significant predictors of facility delivery, the odds of delivering at or on the way to a facility among women in the SP+ group were double the odds among women in the control group (Table 5).

**Table 5:** Association between smartphone intervention & facility delivery.

Stratification	Facility delivery* % (N)	Home delivery % (N)	Unadjusted OR** (95% CI)	p-value	Adjusted OR*** (95% CI)	p-value
**Pooled sample**	68.4 (391)	31.6 (181)				
SP+	73.7 (224)	26.3 (80)	1.68 (1.04–2.72)	0.03	1.96**** (1.21–3.19)	0.01
Control	62.3 (167)	37.7 (101)	1		1	

* At a facility or in transit

** Adjusted only for clustering at 3 levels: administrative district, matched pair, and village.

***Adjusted for clustering and significant variables associated with facility delivery.

**** Intracluster correlation coefficient (ρ) = 0.122.

Significant facility delivery predictors retained in the final model are presented in Table 6. Women with at least one previous facility delivery were 3 times more likely to deliver in a facility compared to women with no previous facility deliveries. We found similar odds for women who had 4 or more ANC visits during pregnancy, and for women who lived near a health facility. The odds of facility delivery among non-Christian women were greater compared to the odds among women who identified as Christian. Quality of the nearest health facility was also a significant predictor: for every one-unit increase in quality score, the odds of facility delivery were 1.4 times greater.

**Table 6:** Reduced mixed-effects model retaining significant predictors of facility delivery (N = 572).

Significant predictors retained	Odds Ratio (95% Confidence Interval)
**Intervention group**	
SP+ (smartphone protocol)	1.96 (1.21–3.19)
Control (paper-based protocol)	1
**Previous facility delivery**	
One or more previous facility deliveries	3.10 (1.93–4.97)
No previous facility deliveries	1
**Religion**	
Non-Christian (Muslim or “none”)	1.73 (1.03–2.92)
Christian	1
**ANC Uptake**	
High (4 or more visits)	3.54 (1.84–6.81)
Low (< 4 visits)	1
**Quality of nearest health facility (z-score)**	1.41 (1.01–1.96)
**Distance to Nearest Health Facility**	
Lives near a HF (< median distance)	2.90 (1.48–5.69)
Lives far from a HF (> median distance)	1
Interaction between ANC uptake and distance from nearest health facility	0.31 (0.12–1.21)

The only significant interaction effect was between ANC uptake and distance to the nearest health facility. When data were stratified by ANC uptake, we found that among women who received less than 4 visits, the odds of facility delivery for those living close to a health facility were nearly triple the odds of facility delivery among those living further away (OR = 2.7; CI = 1.4–5.4). Among women who attended more than 4 ANC visits, distance did not have a significant impact on facility delivery (OR = 1.3; CI = 0.5–3.6). Thus, ANC uptake modified the effect of distance on facility delivery.

### Mechanism: How did SP+ lead to increased facility delivery?

We hypothesized that the frequency of CHW home visits might be an intervening factor influencing the observed effect of SP+ on facility delivery. To test this, a binary variable was created for number of home visits by a CHW during pregnancy (as reported by women clients). It is possible that women’s first visit with a CHW may have been a registration event, with limited or no counselling provided. In most cases, we would expect to see more counselling occur from the second visit onwards, therefore we binned this variable into two categories: a) visited less than twice; or b) visited two or more times.

A simple cross-tabulation between intervention group and reported number of visits from a CHW during pregnancy (< 2 visits or 2 or more visits) showed that women in the SP+ group were significantly more likely to receive 2 or more visits from a CHW compared to those in the control group (72% vs. 60%; p < 0.01). This holds true when controlling for clustering and other factors from the model in Table 6. When the model is run with number of CHW visits as the dependent variable, study group remains a highly significant predictor of CHW visits, and all other predictors become insignificant. This suggests a strong co-linearity between study group and frequency of CHW visits.

Since smartphone use was associated with more frequent household visits by CHW, and with an increased likelihood of facility delivery, we can infer that increased frequency of CHW visits may be an impact pathway through which the SP intervention resulted in higher facility delivery rates.

### Where did SP+ have the greatest impact?

Past facility delivery is inherently linked to parity because first time mothers will have never used facility-based ANC or delivery services. While parity prior to the intervention (i.e. whether a woman was primiparous vs. multiparous at baseline) dropped from the final model, we anticipated that the combination of parity and previous facility delivery might be an important predictor of delivery location. Furthermore, given the known association between antenatal care uptake and facility delivery [43], we hypothesized that ANC uptake might interact with these variables. To explore relationships between these antecedent factors, we grouped participants into three “high risk” groups for further analysis: 1) primiparae with low ANC uptake (< 4 visits); 2) multiparae with no previous facility delivery, and low ANC uptake; and 3) multiparae who reported at least one previous facility delivery and had low ANC uptake.

**Fig 3:**
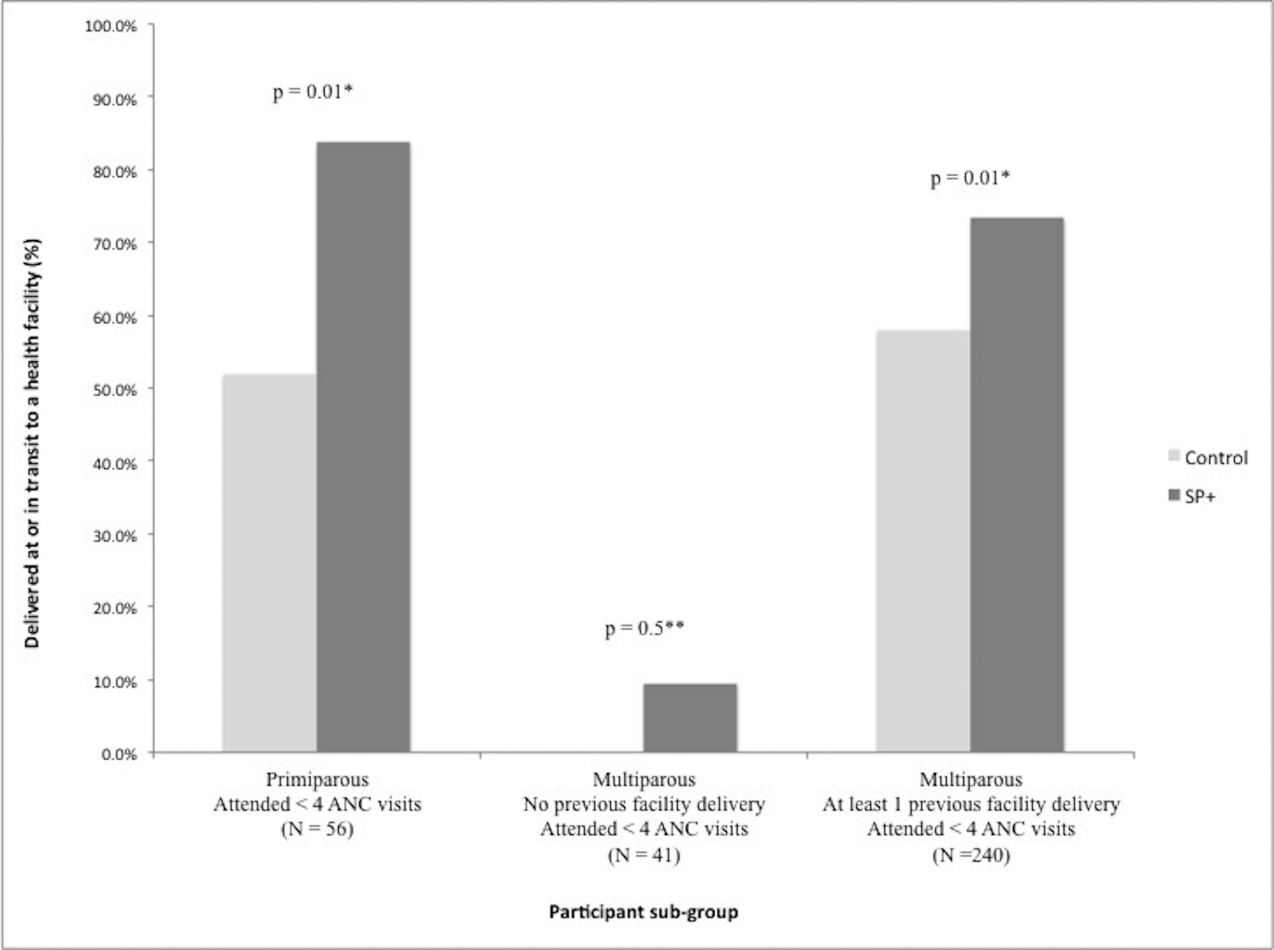
Facility delivery in each study group, stratified by parity, previous facility delivery, and ANC uptake. *Significance assessed by Chi-square tests for proportions. **Significance assessed by Fisher’s exact test for proportions.

Of these three groups, SP+ appears to have the greatest impact on facility delivery among primiparous women who attended less than 4 ANC visits (Fig 3). In this cohort, 84% of women in the intervention group delivered at or in transit to a health facility, compared to only 52% of women in the control group. Similar results were observed among multiparous women who attended fewer than 4 ANC visits. This suggests that smartphone counseling by CHW increased the likelihood of facility delivery in these two groups, even in the absence of high ANC uptake.

## Discussion

This study evaluated the impact of a smartphone decision support application for CHW on their female clients’ use of facility delivery, a known determinant of maternal and newborn survival [3–5]. Among women in the intervention group, the odds of facility delivery were double the odds among women living in control villages, even after accounting for other known predictors. Increased frequency of prenatal visits by CHW in the intervention group may have contributed to the observed effect. Independent factors associated with facility delivery included quality of the nearest health facility, previous facility delivery, uptake of antenatal care, distance to the nearest health facility, and unmeasured socio-cultural factors linked to maternal religious identity. While exposure to the mHealth intervention had a positive impact, having four or more antenatal care visits during pregnancy, previous facility delivery, and living close to a facility were even stronger predictors of facility delivery.

MHealth interventions to improve maternal and child health are increasingly common in low-income countries [17, 19], yet very few studies have used randomized designs to evaluate their impact. Only one other trial, an evaluation of CARE’s Ananya Program in Bihar, India, measured the impact of mobile phone-based tools for frontline workers on maternal and reproductive health outcomes [30]. These authors found no significant impact on facility delivery because it was already very high at baseline, but reported significant impacts on modern contraceptive use, antenatal care uptake, consumption of iron-folate tablets during pregnancy, and several birth preparedness practices that facilitate facility delivery.

In a cell phone intervention trial (“Wired Mothers”) in Zanzibar, Tanzania, pregnant women received health education and appointment reminders via unidirectional SMS (text) messages, as well as vouchers to be used for direct two-way communication with primary healthcare providers [29]. Authors found a significant increase in skilled delivery among urban but not rural women, which was attributed to limited mobile phone access and low receptiveness of the intervention in rural areas. In contrast to the Wired Mothers study, the SUSTAIN intervention was implemented exclusively in rural or periurban areas, and because the application was built as a job aid for CHW, it did not require cell phone ownership by female clients. Taken together, results of the two studies suggest that alternative smartphone functionalities (i.e. job aids for CHW as opposed to direct patient communication) may be particularly useful in remote rural settings, where poverty is highest and facility delivery rates tend to be lower.

Reported previous facility delivery was one of the strongest predictors of delivery location in this setting. Among women reporting at least one previous facility delivery, the odds of facility delivery were about triple the odds among women who had only delivered at home in the past. This pattern is consistent with the plausible expectation that a woman is much more likely to go back for future obstetric care if she first delivered at a facility, especially if her experience was positive. In contrast, a woman’s perceptions of risk associated with childbirth are likely to be lower and her preference for home birth higher if she has delivered safely at home in the past [33, 44]. It is plausible that SP+ may be most effective for first time mothers or those with more exposure to facility-based obstetric services, because they may be younger and have higher perceptions of obstetric risk.

Secondary analyses are also consistent with a working hypothesis that SP+ may boost facility delivery even in the absence of high ANC uptake, particularly among first-time mothers. Fig 3 shows that SP+ had a large impact on first-time mothers with low ANC uptake; a significantly higher proportion (32% more) exposed to SP+ delivered at or on the way to a health facility. The difference of proportions was in the same direction among multiparous women who had previously delivered at a facility after low ANC uptake (15% more) and also among women who had never delivered at a facility (10%, though sample size is inadequate for formal statistical tests). These findings lend weight to recent calls to target safe motherhood interventions according to parity in rural Tanzania [33], and recognize the critical influence of past delivery location on women’s choices. In particular, standalone mHealth interventions may be less effective at changing facility delivery uptake among women who have safely delivered at home in the past.

Findings are in line with previous studies reporting correlations between facility delivery and the distance to the nearest health facility [43, 45–47]. The fact that a higher proportion of women in control villages lived closer to a health facility may have diluted the measured impact of SP+ in this study. Interestingly however, distance from the nearest hospital was not a significant predictor in our model, which conflicts with a recent study on predictors of facility delivery in Tanzania [44]. This may reflect the method used to capture distance–i.e. difference between GPS coordinates–an “as the crow flies” measure, which does not account for actual routes taken or mode of transport, and therefore time and cost required for travel. Alternatively, it is possible that for women who have had several recent interactions with the healthcare system (i.e. those with high ANC uptake), distance is not an insurmountable obstacle because they have made the journey previously. The finding that ANC uptake modified the effect of distance on facility delivery supports this hypothesis. This points to the resourcefulness of women in this context: if they intend to access facility-based services, then they will likely find a way to get there, regardless of distance.

In sum, while mHealth is not a panacea, it can be seen as a health systems strengthening tool, a way to enhance implementation of proven interventions, and a strategy that may diminish the "silo" approach to service/program delivery. Results suggest that mHealth tools for frontline health workers can be used to augment the delivery of existing evidence-based MNCH interventions. However, their potential will only be realized if they are part of a broader, functional health system of integrated programs focused on equitable, and accessible high-quality service delivery for all.

### Study limitations & strengths

Study findings should be interpreted with caution because the study had several limitations and the measured associations, while consistent with direct causation, can have other interpretations. First, we did not capture data on all known facility delivery predictors, thus residual confounding is likely. For example, other studies have shown associations between facility delivery and the presence of traditional birth attendants, tribe/ethnic group, and use of community health insurance and other village-dependent factors (48). Furthermore, several known predictors of facility delivery (e.g. socioeconomic status and maternal education) were not significant and therefore dropped from the final model. The variance in the values of these variables was small, since the women sampled had very similar wealth and education profiles. It is possible that socioeconomic status and maternal education effects may be more evident in a larger, more diversified sample, or if the variables had been measured differently.

Second, it is possible that CHW who received smartphones were initially more thoughtful, attentive or motivated in their work, as a direct result of having a new tool. Consequently, some of the measured effect on client behavior could diminish over time and may not be directly attributable to the sustained functional effects of the smartphones. Independent ‘novelty effects’ [17] were not separately tested in this study, therefore questions remain about the long-term sustainability of such tools for CHW. Future research should attempt to tease apart such effects to assess whether measured impacts diminish, improve or remain unchanged over time.

Third, because the evaluation was conducted in a “real world” programmatic context, it was not feasible to mask intervention allocation. CHW in SP+ and control areas often interact through various seminars and training opportunities, and those in the control group may have been demotivated when they did not receive a smartphone. Third, the analysis was not based on “difference-in-differences” comparison of facility delivery rates between intervention arms and may have been strengthened if baseline comparison measures were available. However, we have no reason to believe that baseline facility delivery rates were different in SP+ villages compared to control villages, and this potential drawback was likely addressed during the randomization stage.

Although the study may have been strengthened by these and other potential improvements, the interpretations are based on a strong cluster randomized design and careful analytic attention to potential pathways for impact. Finally, the study was further strengthened by statistical adjustment for clustering effects at multiple levels.

### Conclusions

Training of CHW on smartphone application use appears to add value through more frequent household visits and may be efficacious as one component of intervention packages designed to increase uptake of safe facility delivery. Within the context of this study, indications are that smartphone-based support for CHW achieved the highest impact when used during counselling with first-time mothers, and particularly helped to boost facility delivery rates among women with low ANC uptake. This study provides some evidence that such applications might improve the effect of CHW counseling on facility delivery. These positive indications provide a rationale for investing in further research to evaluate cost effectiveness, sustainability, and replicability in other contexts.

## Supporting information

S1 Checklist (PDF)

S1 Table (PDF)

S1 Protocol (PDF)

S1 Questionnaire (PDF)

S2 Questionnaire (PDF)

S1 Supporting Information (PDF)
