# Supplementary material for: Impact of smartphone-assisted prenatal home visits on women’s use of facility delivery: Results from a cluster-randomized trial in rural Tanzania
Source: PLoS One. 2018 Jun 18;13(6):e0199400. doi: 10.1371/journal.pone.0199400 (PMC6005474; doi:10.1371/journal.pone.0199400)
Supplement: S1 Table — (PDF) [file pone.0199400.s002.pdf]

Table 1. SUSTAIN-1 Trial secondary outcome measures. Data for all outcomes were assessed by retrospective report at interview, after delivery*

Secondary outcome	Description	Timing and method of assessment
Gestational age at first antenatal care (ANC) visit	Number of months into pregnancy at the time of participant's first ANC visit	
Iron supplementation during pregnancy	Dichotomous: Did the participant take 100 IFA tablets during pregnancy?	By retrospective report at interview, after delivery
HIV screening	Dichotomous: Was the participant tested for HIV during pregnancy?	By retrospective report at interview, after delivery
De-worming	Dichotomous: Did the participant take de-worming medication during pregnancy?	By retrospective report at interview, after delivery
Bed net use	Dichotomous: Did the participant sleep under a bed net during pregnancy?	By retrospective report at interview, after delivery
Malaria prophylaxis	Dichotomous: Did the participant receive two doses of IPTp during pregnancy?	By retrospective report at interview, after delivery
Tetanus vaccination	Dichotomous: Did the participant receive two TT vaccinations during pregnancy?	By retrospective report at interview, after delivery
Number of referrals per CHW	Discrete: How many women were referred to a clinic during pregnancy by each CHW?	By retrospective report at interview, after delivery
Referral uptake	Continuous: Of the women who were referred, what proportion actually sought care from a clinic?	By retrospective report at interview, after delivery
Presence of partner at ANC	Dichotomous: Was the participant accompanied by her spouse/partner for at least one ANC visit?	By retrospective report at interview, after delivery
Presence of partner at delivery	Dichotomous: Was the participant accompanied by her spouse/partner during delivery?	By retrospective report at interview, after delivery
Birth plan	Dichotomous: Did the participant develop a birth plan in advance?	By retrospective report at interview, after delivery
Early initiation of breastfeeding	Dichotomous: Did the participant initiate breastfeeding within 1 hour of birth?	By retrospective report at interview, after delivery
Exclusivity of breastfeeding at 7 days	Dichotomous: Was the infant breastfed exclusively during the	By retrospective report at interview, after

	first week after birth?	delivery
BCG vaccination	Dichotomous: Was the infant given BCG vaccination within the first week after birth?	By retrospective report at interview, after delivery
Maternal knowledge of danger signs during pregnancy	Discrete: Number of pregnancy danger signs listed by participant	By retrospective report at interview, after delivery
CHW efficiency	Total number of participant/infant dyads tracked, as a proportion of the total number of dyads each CHW is responsible for	By retrospective report at interview, after delivery
