# Supplementary material for: Impact of smartphone-assisted prenatal home visits on women’s use of facility delivery: Results from a cluster-randomized trial in rural Tanzania
Source: PLoS One. 2018 Jun 18;13(6):e0199400. doi: 10.1371/journal.pone.0199400 (PMC6005474; doi:10.1371/journal.pone.0199400)
Supplement: S1 Protocol — (PDF) [file pone.0199400.s003.pdf]

UNIVERSITY OF TORONTO

Office Use Only<br>Protocol Number:

Office of the Vice-President, Research and Innovation

Office of Research Ethics

ETHICS REVIEW PROTOCOL SUBMISSION FORM FOR SUPERVISED AND SPONSORED RESEARCHERS

(For use by graduate students, post-docs, and visiting professors/researchers)

SECTION A – GENERAL INFORMATION

1. TITLE OF RESEARCH PROJECT

Evaluation of an mHealth Intervention to Improve Women's access to Maternal Health Services in Rural Tanzania

2. INVESTIGATOR INFORMATION

Investigator:

Title (e.g., Dr., Ms., etc.): Ms	Name: Kristy Hackett
Department (or organization if not affiliated with U of T): Dalla Lana School of Public Health	
Mailing address: 6th floor, 155 College St, Toronto, ON M5T 3M7	
Phone: 647-882-9908	Institutional e-mail: kristy.hackett@mail.utoronto.ca

Level of Project:

Student Research:	Doctoral ☒	Masters ☐
Post-Doctoral Research ☐	Visiting professor/researcher ☐	Course Based ☐
CBR/CBPR ☐	Other ☐ (specify:)	

Faculty Supervisor/Sponsor:

Title: Professor	Name: Daniel Sellen
Department (or organization if not affiliated with U of T): Anthropology, Nutrition, Public Health Sciences	
Mailing address: 19 Russell Street, Toronto M5S 2S2	
Phone: 416-978-8112	Institutional e-mail: dan.sellen@utoronto.ca

Co-Investigators:

Are co-investigators involved? Yes ☒ No ☐

Title: Professor	Name: Sebalda Leshabari
Department (or organization if not affiliated with U of T): Muhimbili University of Health & Allied Sciences, Department of Nursing	
Mailing address:	
Phone:	Institutional e-mail: seolesh@yahoo.com

Title:	Name:
Department (or organization if not affiliated with U of T):	
Mailing address:	
Phone:	Institutional e-mail:

Please append additional pages with co-investigators' names if necessary.

3. UNIVERSITY OF TORONTO RESEARCH ETHICS BOARD:

Health Sciences ☒ Social Sciences, Humanities and Education ☐ HIV/AIDS ☐

To determine which Research Ethics Board (REB) your protocol should be submitted, please consult: http://www.research.utoronto.ca/for-researchers-administrators/ethics/human/boards-committees/..

4. LOCATION(S) WHERE THE RESEARCH WILL BE CONDUCTED:

If the research is to be conducted at a site requiring administrative approval/consent (e.g., in a school), please include all administrative consent letters. It is the responsibility of the researcher to determine what other means of approval are required, and to obtain approval prior to starting the project.

University of Toronto ☐
Hospital ☐ specify site(s)
School board or community agency ☐ specify site(s)
Community within the GTA ☐ specify site(s)
International ☒ specify site(s) Singida Region, United Republic of Tanzania
Other ☐ specify site(s)

The University of Toronto has an agreement with the Toronto Academic Health Sciences Network (TAHSN) hospitals regarding ethics review of hospital-based research where the University plays a peripheral role. Based on this agreement, certain hospital-based research may not require ethics review at the University of Toronto. If your research is based at a TAHSN hospital please consult the following document to determine whether or not your research requires review at the University of Toronto. http://www.research.utoronto.ca/for-researchers-administrators/ethics/human/at-a-glance/where-to-apply-tahsn-institutions/

5. OTHER RESEARCH ETHICS BOARD APPROVAL(S)

(a) Does the research involve another institution or site? Yes ☒ No ☐
(b) Has any other REB approved this project? Yes ☒ No ☐
If **Yes**, please provide a copy of the approval letter upon submission of this application.
If **No**, will any other REB be asked for approval?
Yes ☐ No ☐
Please note that REB approvals from other sites must be submitted to the ORE at U of T

6. FUNDING OF THIS PROJECT

(a)

Funding Status	Source and Type	Details
Funded ☒	Agency: CIDA	Fund #: 493309 (6 digits)

	Agency:	Fund # :4 (6 digits)
Applied for funding ☐	Agency:	Submission date:
	Agency:	Submission date:
Unfunded ☐<br>If unfunded, please explain why no funding is needed:		

(b) If one protocol is to cover more than one grant, please include all fund numbers:

7. CONTRACTS

Is this research to be carried out as a contract? Yes ☒ No ☐

If yes, is there a University of Toronto funding or non-funded agreement associated with the research? Yes ☒ No ☐

If **Yes,** please append a copy of the agreement with this application.

Is there any aspect of the contract that could put any member of the research team in a potential conflict of interest? Yes ☐ No ☒

If yes, please elaborate under #10.

8. PROJECT START AND END DATES

Estimated start date for the component of this project that involves human participants or data: April 15, 2013

Estimated completion date of involvement of human participants or data for this project: January 31, 2015

9. SCHOLARLY REVIEW:

(Please note: for submissions to the **HIV REB** from community investigators, scientific review is a pre-requisite for ethics review. If your study is unfunded, please contact the OHTN to arrange a scientific review prior to completing your ethics submission.)

(a) Please check one:

I. ☒ The research has undergone scholarly review by thesis committee, departmental review committee, peer review committee or some other equivalent (Specify review type – e.g., departmental research committee, supervisor, CIHR, SSHRC, OHTN, etc.): Supervisor

II. ☐ The research will undergo scholarly review prior to funding (Specify review committee – e.g., departmental research committee, SSHRC, CIHR peer-review committee, etc.):

III. ☐ The research will not undergo scholarly review (Please note that all research greater than minimal risk requires scholarly review)

(b) If box I or II above was checked, please specify if:

☒ The review was specific to this protocol

☐ The review was/will be part of a larger grant

10. CONFLICTS OF INTEREST

(a) Will the researcher(s), members of the research team, and/or their partners or immediate family members:

(i) Receive any personal benefits (e.g., financial benefit such as remuneration, intellectual property rights, rights of employment, consultancies, board membership, share ownership, stock options, etc.) as a result of or in connection with this study? Yes ☐ No ☒

(ii) If **Yes**, please describe the benefits below. (Do not include conference and travel expense coverage, or other benefits which are considered standard for the conduct of research.)

Not applicable.

(b) Describe any restrictions regarding access to or disclosure of information (during or at the end of the study) that have been placed on the investigator(s). These restrictions include controls placed by the sponsor, funding body, advisory or steering committee.

Not applicable.

(c) Where relevant, please explain any pre-existing relationship between the researcher(s) and the researched (e.g., instructor-student; manager-employee; clinician-patient; minister-congregant). Please pay special attention to relationships in which there may be a power differential – actual or perceived.

No formal relationship exists between myself and the proposed research participants.

(d) Please describe the decision-making processes for collaborative research studies. If Terms of Reference exist, attach them. Collaborative research studies include those where a number of sites (e.g. other universities, non-TAHSN hospitals, etc.) are involved, as well as those that involve community agencies.

Prof Sellen serves as the primary academic advisor on the research design. Dr. Sebalda Leshabari serves as secondary advisor and local supervisor. Dr. Leshabari, Prof Sellen and K. Hackett will participate in regular bi-weekly conference calls to discuss all matters related to the proposed study. All decisions related to the research will be made as a team.

Curtis Lafleur is the Project Manager for the CIDA-funded SUSTAIN[†] project and is based at World Vision Canada, in Mississauga. Curtis will facilitate technical support for the project. We will consult with Curtis on a regular basis and seek his input where necessary.

World Vision Canada and World Vision Tanzania will liaise with Tanzania Ministry of Health and all national, regional, District, Ward and Village governance levels to coordinate collaboration with appropriate administrators, participants and other stakeholders, such as the MHealth technical providers and other SUSTAIN project partners organizations. Terms of Reference are included under the contract appended (Appendix A).

[†] **SUSTAIN stands for 'Supporting Systems to Improve Nutrition Maternal Newborn & Child Health'**

SECTION B – SUMMARY OF THE PROPOSED RESEARCH

11. RATIONALE

Describe the purpose and scholarly rationale for the proposed project. State the hypotheses/research questions to be examined. The rationale for doing the study must be clear. Please include references in this section.

In Tanzania, the provision of appropriate medical care during pregnancy, and the attendance of skilled health personnel during labour and delivery are crucial if maternal, neonatal and child health (MNCH) is currently inadequate in all administrative Districts. Ensuring access to skilled health professionals during pregnancy and childbirth remains difficult to achieve due to funding shortages and gaps in human resources for health (1). To overcome these challenges, currently available human resources must be identified and utilized in the most effective and efficient ways possible. Frontline community health workers (CHWs) can be trained to address gaps in MNCH services through delivery of basic medical care and promotion of healthy behaviours. Specifically, CHWs can facilitate health behaviour change to improve women's utilization of antenatal care and skilled, facility-based delivery.

Despite the potential of CHWs to improve MNCH, issues with equity of service delivery, incentivization, CHW attrition, record keeping and information management are persistent challenges (2). In the absence of sufficient training, supervision and job aids, CHW performance can be suboptimal, characterized by high error rates and low protocol compliance (3). A lack of systematic tools for tracking and monitoring clients prevents CHWs from following up and referring critical cases to health professionals in a timely manner (4). Innovative strategies are urgently needed to improve the quality of care provided by CHWs.

The use of Mobile health (mHealth) technologies can provide affordable solutions to many of these challenges. Interventions combining the use of CHW services and mHealth applications have the potential to synergistically improve MNCH. However, while mHealth is considered a promising approach, it is still an emerging field and thus requires a stronger evidence base. There is currently a need for more rigorous scientific evaluations of mHealth interventions (6,7).

The *overall objective* of this study is to investigate whether a novel mHealth application can be used by CHWs to increase women's access to maternal health services in the Singida region of rural Tanzania. Specific *research questions* are as follows:

1. What is the impact of the mHealth intervention on women's antenatal care attendance and facility-based delivery?

2. What is the impact of the mHealth intervention on women's knowledge of danger signs during pregnancy, and other prenatal and neonatal health indicators[‡]?

3. How do qualitative measures of job satisfaction and perceived levels of self-efficacy among CHWs differ depending on mHealth application use?

4. How does the mHealth application influence perceived quality of care among CHWs' female clients?

[‡] ***A full list of indicators to be examined can be found in Table 1, Appendix B***

5. What are the barriers and facilitators of maternal health services use, and how does mHealth influence these factors?

References:

1. Kerber KJ, de Graft-Johnson JE, Bhutta ZA, Okong P, Starrs A, Lawn JE. Continuum of care for maternal, newborn, and child health: from slogan to service delivery. Lancet 2007;370(9595):1358-69.

2. Bhutta ZA, Lassi ZS, Pariyoand G, Huicho L. Global Experience of Community Health Workers for Delivery of Health Related Millennium Development Goals: A Systematic Review, Country Case Studies, and Recommendations for Integration into National Health Systems: World Health Organization; 2010.

3. Florez-Arango JF, Iyengar MS, Dunn K, Zhang J. Performance factors of mobile rich media job aids for community health workers. Journal of the American Medical Informatics Association: JAMIA 2011;18(2):131-7.

4. Haines A, Sanders D, Lehmann U, Rowe AK, Lawn JE, Jan S, et al. Achieving child survival goals: potential contribution of community health workers. Lancet 2007;369(9579):2121-31.

5. Gurman TA, Rubin SE, Roess AA. Effectiveness of mHealth Behavior Change Communication Interventions in Developing Countries: A Systematic Review of the Literature. Journal of Health Communication: International Perspectives 2012:17(1):82-104.

6. Free C, Phillips G, Watson L, Galli L, Felix L, et al. The Effectiveness of Mobile-Heath Care Service Delivery Processes: A Systematic Review and Meta-Analysis. PLoS Med 10(1): e1001363.

7. Free C, Phillips G, Galli L, Watson L, Felix L, et al. (2013) The Effectiveness of Mobile-Health Technology-Based Health Behaviour Change or Disease Management Interventions for Health Care Consumers: A Systematic Review. PLoS Med 10(1): e1001362.

12. METHODS

(a) Please describe all formal and informal procedures to be used. Describe the data to be collected, where and how they will be obtained and how they will be analyzed.

Study Design: We will employ a *concurrent embedded mixed-methods design* to evaluate the process and impact of an mHealth intervention (a cluster randomized controlled trial) implemented by World Vision Tanzania. A description of the mHealth application and intervention design can be found in Appendix C.

It should be noted that we are seeking ORE approval for the evaluation component only. While we did provide scientific inputs on the intervention design, with reference to the Ottawa Statement on cluster controlled trials, we are not seeking ORE approval for implementation of the mHealth intervention itself.

Quantitative methods will be used to answer research questions 1 and 2. We will take advantage of the mHealth intervention's *cluster randomized controlled trial design* to measure the impact of the mHealth intervention on quantitative access indicators such as women's antenatal care attendance,

facility-based delivery, knowledge of danger signs during pregnancy, and a number of other clinical outcomes (Table 1, Appendix B).

Objectives 3, 4 and 5 will be explored using mixed methods, at several data collection points throughout the intervention period (described below).

Quantitative components:

There are three quantitative components in the evaluation study:

A) A brief self-administered survey will be given to CHWs at the time of enrolment. Data will be collected from participants in both arms of the study. The survey can be found in Appendix D.

B) Programmatic data, routinely collected by CHWs:

Most quantitative outcomes for pregnant women and mothers (Table 1, Appendix B) are routinely collected by CHWs as part of regular WV programming. At the end of the intervention period, a research assistant will be responsible for extracting the data from de-identified client registers and entering this information into a study database. Secondary analysis of this programmatic data will be conducted with the permission of World Vision Tanzania. Data will be collected for consenting participants in both arms of the study.

C) A research team will administer semi-structured household surveys to a randomly selected sub-sample of women (CHW clients) at the end of the intervention period. These household visits will take place at between 0 - 7 months post-delivery, depending on the gestational age of mothers at the beginning of the intervention period. Data will be collected for participants in both arms of the study. The semi-structured household survey for women clients can be found in Appendix G.

We will use electronic tablets to administer the household surveys. We have pre-tested both paper-based and electronic versions of the household survey and found that the tablets are most efficient, user-friendly, lead to fewer errors/ambiguities in data collection, and are widely accepted by women in rural areas.

Tablets are equipped with GPS capabilities and thus will be used to capture location coordinates at the end of each interview. The benefit of collecting and analyzing GPS data is that it will provide a more accurate and reliable measure of distance from women's homes to the nearest health facility, a potentially important confounding factor in this study. In our survey we ask participants how long it takes them to travel to the nearest health facility. While this measure could be used as a proxy for distance, we suspect that participants' estimations may be subjective and unreliable. GPS data will be used to determine more precise distances, leading to better results and more meaningful recommendations for policy and programming.

All data collected with tablets (including GPS data) will be de-identified; only anonymous participant ID codes will be used. At the end of each research day, data will be synced over wifi to a secure password-protected Google server then manually transferred to an encrypted/password-protected data file. Once the data is transferred to the server, it will be cleared from the tablet's storage folder.

D) A Health Facility "Checklist" will be used to evaluate the quality of health facilities in the study area (N = 14). The checklist survey can be found in Appendix K.

SPSS software on KH's computer will be used to analyze quantitative data.

Qualitative/Mixed-Methods Component:

A mix of Qualitative and/or quantitative methodologies will be employed to explore the following domains: 1) levels of job satisfaction, and perceived benefits of and challenges to, mHealth application use among CHWs; and 2) female clients' perceptions of application effectiveness and quality of CHW care, and 3) perceived barriers to maternal health service access, as reported by CHWs, CHW clients, and health facility staff. Target groups will be randomly selected sub-samples from the larger quantitative study, thus participants in the qualitative research will undergo an additional consent process and sign a separate informed consent form. Consent to participate in the quantitative component of the study does not imply consent to participate in the qualitative component.

Target Group 1: CHWs

There will be three phases of qualitative research with CHWs.

A) We will conduct a process evaluation of CHWs' mHealth training activities. This will involve non-participatory observations and informal discussions during the training period (August 2013). To understand the training process and any associated challenges, KH will attend the CHWs' mHealth training workshop (approximately 1 week in duration). During this workshop CHWs will be trained on application use by D-Tree, the mHealth technology provider. If necessary, data collected will be used to modify qualitative data collection tools, to be used in B) and C) below. Any modifications to these tools will be submitted to the ORE as an amendment.

B) a. Semi-structured one-on-one interviews will be conducted with all 32 CHWs in the experimental group, and 32 randomly selected CHWs in the control group (total N = 64). These interviews will be conducted approximately 6-8 weeks after the mHealth training session. According to our experienced colleagues at D-Tree, it should take the CHWs several months to get used to using the mHealth application. Therefore, interviewing CHWs 6-8 weeks into the intervention will allow us to capture CHWs' experiences after they've had ample time to adapt to using the mHealth application with clients. Transcripts of the interviews with participants in the two arms of the study will be compared and analyzed for differences in attitudes and perceptions.

The semi-structured interview guide for CHWs can be found in Appendix H.

b. We will re-assess the same CHWs' perceived self-efficacy, confidence and job satisfaction near the end of the intervention period (estimated May 2014). This will allow us to capture any changes in these measures over time. Only the first section of the interview guide (likert-scale questionnaire) will be administered at this time.

C) Two follow-up focus groups (one in each study arm) will also be conducted near the end of the evaluation period. These focus group discussions will serve a validation piece (Corbin & Strauss, 2008) and will allow for follow-up on issues that surfaced previously during the interviews. The key domains to be investigated during both interviews and focus groups with CHWs are as follows:

- job satisfaction
- satisfaction with mHealth tool, specifically (mHealth/experimental group only)
- perceived self-efficacy (confidence and competence with respect to duties and responsibilities)
- perceived challenges/barriers and benefits/facilitators of the job
- perceived attitudes of clients (mothers, partners, relatives, etc.)

Target Group 2: Mothers (CHWs' female clients)

A) A sub-sample of ~24 female clients will be randomly selected from each study arm (total N = 48) to participate in focus group discussions (FGDs). We will conduct a total of 8 FGDs with CHW clients (4 in each of the 2 catchment areas). The aim of these FGDs will be to explore the following:

- Women's perceived quality of care provided by CHWs
- Reasons for choosing facility and/or home births (if applicable)
- Perceived barriers to ANC/obstetric services access
- Attitudes towards traditional birth attendants (CHWs), CHWs, heath facility staff
- Perceived unmet healthcare needs

Through comparison of discussions with women who interact with CHWs in the experimental group to those who interact with CHWs in the control group, we will explore how mHealth might influence perceived quality of care among clients.

The FGD guide for women clients can be found in Appendix I.

B) Four follow-up focus group discussions will be held with a sub-sample of women in each study arm near the end of the intervention period (between 10-12 months). These focus group discussions will serve as a validation piece (Corbin & Strauss, 2008) and will allow for follow-up on issues that surface previously during interviews.

**Please note that the follow-up FGD guides for both CHWs and female clients will be developed following preliminary analysis of the first round of qualitative data collection. Results of the initial interviews and focus groups will inform the development of follow-up tools. These tools will be submitted to the ORE as an amendment to be approved prior to use.*

Target Group 3: Health facility staff (nurses, medical assistants, clinical officers)

a. We will conduct 14 semi-structured interviews with health facility staff from dispensaries in both the experimental and control areas. These staff will be supervisors for CHWs. The goal of these interviews will be to capture supervisors' impressions regarding women's ANC attendance, facility-based delivery and for the mHealth/experimental arm, whether they feel the intervention is contributing to changes in clinic attendance. We will also assess the supervisors' opinions regarding CHW performance and quality of care provided by the CHWs. Comparing supervisors' assessments of CHW performance in control vs. experimental villages will help us to determine whether the mHealth intervention is working.

The interview guide for facility staff can be found in Appendix J.

b. We will re-assess the same supervisors' opinions regarding CHW performance and quality of care near the end of the intervention period (May 2014). This will allow us to capture any changes in these measures over time. Only the first section of the interview guide (likert-scale questionnaire) will be administered at this time.

Collection of data for potential confounding factors:

We have identified health facility quality as a potentially important source of confounding in this study. For example, women's decisions to utilize maternity services will likely depend on various

facility-level factors that we do not have control over in this study. Such "fixed" variables include: distance to closest facility, staffing of facility, level of training received by facility staff, quality of services provided, and stock levels of essential medical supplies/equipment at each facility. In order to control for these variables during analysis, we have developed a multivariate health facility "scorecard". This tool was developed with inputs from World Vision staff and other local colleagues working in community health. Assessments of health facilities will be made during facility visits, following interviews with CHW supervisors.

The Health Facility Assessment tool can be found Appendix K.

Data Collection procedures:

KH will train a team of experienced research assistants (fluent in both English and Swahili) to lead/facilitate all qualitative interviews and focus groups. Previous research on women's reproductive experiences in rural Tanzania reported that the presence of Caucasian North American-based researchers attracted onlookers, thereby compromising privacy and influencing participant responses (Haws et al. 2010). So as to avoid compromising the privacy of interviews and focus groups and the responses of participants, KH will focus her efforts on training a team of experienced research assistants to collect data for this study. Following each interview or focus group, KH will lead a debriefing meeting with interviewers/facilitators to ensure that data is collected according to protocol and that appropriate techniques (probing, judging tone of voice etc.) have been used. While KH may be present for some interviews as a quality control measure, this will be documented and the data will be analyzed accordingly.

All interviews and FGDs will be recorded with the permission of participants. The information will then be transcribed in Kiswahili by a research assistant then translated into English. In order to maintain confidentiality and anonymity of research participants throughout this process, no identifying information will be included in Kiswahili or English transcripts.

English transcripts will be imported to Nvivo software on KH's personal computer for thematic content analysis.

(b) Attach a copy of all questionnaires, interview guides and/or any other instruments.

(c) Include a list of appendices here for all additional materials submitted (e.g., Appendix A – Informed Consent; Appendix B – Interview Guide, etc.):

Appendix A – Terms of Reference for Collaboration (U of T and World Vision)
Appendix B – List of quantitative outcome variables to be measured (Table 1)
Appendix C – Description of World Vision's mHealth intervention (a cRCT)
Revised: Appendix D – Informed Consent forms for community health workers
Appendix E – Informed Consent forms for women (CHW clients)
Appendix F – Confidentiality form for research assistants
Revised: Appendix G – Household Survey for Women (CHW clients)
Revised: Appendix H – Semi-structured interview guide for CHWs
Revised: Appendix I – Focus Group Discussion guide for women clients
Revised: Appendix J – Semi-structured interview guide for health facility staff
Appendix K – Health Facility Assessment Tool ("Checklist")
Appendix L – REB Approval Letter from Tanzania National Institute for Medical Research;
Research Permit from Tanzania Commission for Science & Technology

13. PARTICIPANTS AND/OR DATA

(a) Describe the participants to be recruited, or the individuals about whom personally identifiable information will be collected. List the inclusion and exclusion criteria. Where the research involves extraction or collection of personally identifiable information, please describe from whom the information will be obtained, what it will include, and how permission to access the data is being sought. (Strategies for recruitment are to be described in section #15.) Where applicable, justify the sample size.

Participants to be recruited: The target population consists of 1) CHWs; 2) women to whom CHWs provide health services (clients); and 3) health facility staff (CHW supervisors) from each district.

Inclusion/exclusion criteria for CHWs:
Potential CHW participants must be in one of the two catchment areas of the World Vision mHealth intervention (Mtinko or Kisiriri wards) and b) must have completed the national, standardized three-week Ministry of Health training course on Maternal Newborn & Child Health for CHWs. There are no exclusion criteria for CHWs; participants may be male or female and can be any age.

In study areas, each village typically has one male and one female CHW. Both male and female CHWs will be included in the study because we would like to capture the most realistic picture of how the mHealth intervention operates. We will enroll equal numbers of male and female CHWs so that we can identify any differences in intervention uptake, performance and job satisfaction between the two groups.

We have considered the possibility that clients may respond and interact differently with male vs. female CHWs, however because we will be working with existing CHWs, this is beyond the control of researchers. We will explore differences in perceived quality of care provided by male vs. female CHWs through qualitative data collection with female clients.

Inclusion/exclusion criteria for women (CHW clients):
Potential women participants must fit the following inclusion criteria: a) between the ages of 16 – 49 years; b) client of one of the CHWs enrolled in the mHealth study; c) visited at least once by a CHW following the i-MNCH/mHealth training, and during their most recent pregnancy; and e) the most recent delivery was a live birth and the child is still living.

There are no exclusion criteria for women.

Inclusion/exclusion criteria for health facility staff: Health facility staff must work full-time in a dispensary or health centre within one of the two WV catchment areas and must be a designated supervisor of one or more CHWs in the study. There are no exclusion criteria for health facility staff.

Sample Size justification:

Required sample size was estimated using the methods outlined by Campbell et al. (2004) (13). In this paper, the authors emphasize the importance of accounting for the effect of clustering on sample size. This can be achieved statistically by using an appropriate intra-cluster correlation coefficient (ICC) value, which is based on the ratio of the between- to within-cluster variance for a given outcome of interest. After reviewing the literature for cluster RCTs measuring skilled delivery as a main outcome, it was determined that an appropriate ICC value in this context is 0.14. Using the equation outlined by Campbell et al. and assuming 32 villages (clusters), we will need to enroll a

total of 524 women (262 in each study arm). This sample size will allow us to detect a 10% difference between the experimental and control groups, at a 5% significance level and 80% power. We anticipate a 30% loss to follow-up (women may be travelling or unreachable the day of the interview) and will therefore oversample accordingly. Mothers who consent to participate will be asked to complete a semi-structured household survey at that time, thus no follow-up visit will be necessary.

Assuming the intervention is evaluated in 32 villages (16 control, 16 experimental), a total of 64 CHWs will be eligible to participate (2 CHWs/village). Based on local fertility rates and birth rate estimates, we estimate that on average, each CHW will interact with 1.47 newly pregnant women each month, therefore approximately 88 new pregnancies will be reported each month in the study area. These estimations are based on latest available data provided by the Mtinko World Vision office.

Collection of personally identifiable information:

Baseline surveys will collect the following data for each CHW: name, contact information (phone number if available), age, sex, education, village name, work experience, previous training, and health dispensary affiliation. For pregnant women, semi-structured household surveys will collect the following information: age, parity, education, SES/HH income, history of obstetric complications, village name, proximity to health dispensary, previous place of delivery, and experience with traditional birth attendants.

For both target groups, a research assistant will obtain consent and the data itself from participants directly. All personally identifiable data will be coded so that confidential information is protected and individual anonymity is ensured. Names and codes will be stored in separate password-protected files accessible only by myself and one other research assistant.

Permission to conduct secondary analysis on pregnant women's medical information (programmatic data – collected via CHW registers) will be obtained from each woman directly, by members of the research team, independent of CHWs. On the consent form, there are two separate research components requiring participant consent: 1) participation in household survey; and 2) permission for use of CHW register data for secondary analysis. Thus, women will have the option of participating in both components of the study, or they may choose to participate only in one. Data collectors will explicitly inform women that involvement in the research is completely voluntary, and separate from health care, and that their decision to participate will have no impact on the care received from CHWs. The data received from World Vision by the research team will be completely de-identified. We will only use aggregate data in this part of the analysis. This will also be emphasized during the consent process.

At the end of the intervention period, a member of the research team will be responsible for photocopying/entering data from the de-identified registers into a study database. Information will be entered into a password-protected computer database, and the computer will be password protected and kept in a locked office at the World Vision Singida office. Photocopies will be stored in a locked filing cabinet at World Vision's Singida office during the data entry phase, and then completely shredded once data entry is complete.

All research assistants involved in data collection and management will be asked to obtain an online National Institutes of Health Research Ethics Training certificate, and will be required to sign a confidentiality agreement prior to their participation (Appendix E).

Prior to the collection of data through surveys, interviews and focus groups, all participants will be informed that any information they provide is entirely voluntary and that every effort to maintain confidentiality will be made.

(b) Is there any group or individual-level vulnerability related to the research that needs to be mitigated (for example, difficulties understanding informed consent, history of exploitation by researchers, power differential between the researcher and the potential participant)?

We will assess comprehension of the information provided during the consent process both directly and indirectly. We will first assess comprehension indirectly by verifying with Tanzanian colleagues that the language in consent forms and scripts are appropriate for each group. Modified consent documents will then be assessed directly through pilot testing. Documents will be pre-tested with individuals that are external to the study but within the same demographic group(s). Participants will also be encouraged to ask questions during the consenting process if they do not understand any of the information presented.

Power inequities remain an ongoing challenge in any kind of research with vulnerable groups. However, the training of the primary field researcher and the PI include topics related to gender, power issues, and social inequalities, therefore we will be sensitive to these dynamics and will respond appropriately.

That said, power differentials between the researcher and potential participants will be minimal and based mainly on differences in education levels. CHWs typically have some formal education and are all able to read and write; however education levels among pregnant women in the communities may be minimal. This power differential will be minimized because we will pre-test all consent documents, survey tools and interview guides to ensure the language used is appropriate and comprehensible by participants in each target group. Consent documents will be read to participants orally, and participants will be encouraged to ask questions. Any research assistants involved in data collection and/or the consenting process will undergo training on appropriate conduct and research methodologies.

14. EXPERIENCE OF INVESTIGATORS WITH THIS TYPE OF RESEARCH

(a) Please provide a brief description of previous experience with this type of research by (i) the principal investigator/supervisor or sponsor, (ii) the research team and (iii) the people who will have direct contact with the participants. If there has not been previous experience, please describe how the principal investigator/research team will be prepared.

Kristy Hackett is a PhD candidate in the School of Public Health, Faculty of Medicine, at the University of Toronto. As part of her Masters degree in Medical Anthropology, Kristy managed a research project on an infant and young child-feeding project in Bangladesh, where she worked closely with BRAC, a large Bangladeshi NGO. Kristy has collaborated on a number of global health research projects. For example, she was involved in the secondary analysis of data from a large maternal and child nutrition project in Ghana. Kristy has also been involved in women’s health research in Canada. Most recently, Kristy was a co-investigator in an adolescent reproductive health project at the Hospital for Sick Children in Toronto. Kristy has completed relevant course work in maternal and child health, global health research methods (including research ethics), gender and health, epidemiology, and qualitative data analysis.

Kristy has already made two trips (February and November of 2012) to Tanzania in preparation for this project. During these visits, Kristy conducted formative research and met with WV colleagues to discuss study design and logistics for the project.

Kristy will be responsible for recruiting and training research assistants, collecting data (with the aid of interpreters/translators) and will take a lead in data analysis. Kristy will spend up to 9 months in country throughout the duration of this study. She will be responsible for managing research assistants and liaising with World Vision's SUSTAIN project staff in Singida rural and Iramba. Data collected for this study will be used as partial fulfillment of Kristy's doctoral degree at the University of Toronto.

Dr. Daniel Sellen is a Professor of Anthropology at the University of Toronto, with cross-appointments in the departments of Nutrition Sciences and Public Health Sciences. Prof Sellen also holds a Canada Research Chair in Human Ecology and Public Nutrition. Prof Sellen serves as the primary academic advisor on the research design and will oversee the scientific aspects of the entire study. Prof Sellen has extensive experience in research with mothers and infants in sub-Saharan Africa. KH will remain in regular contact with Prof Sellen in person in Toronto or in Tanzania or by email and Skype throughout the entire research process.

Dr. Sebalda Leshabari is a trained Nurse, and associate dean of Nursing at Muhimbili University. She is also an experienced health researcher with extensive experience working with women and children in Tanzania. Dr. Leshabari will fill the role of local supervisor for this project. Dr. Leshabari will contribute to the development of all protocols and study tools. She will also ensure that implementation of the study adheres to the requirements of Tanzania's National Institute for Medical Research (NIMR). Dr. Leshabari will also oversee the project by meeting regularly with K. Hackett, either in person or remotely. She will also provide key inputs during the data analysis phase. Dr. Leshabari will also be involved in the development of publications.

Research Assistants

A research team will be recruited and trained beginning in April of 2013. These individuals will all have a post-secondary degree, will have previous research experience, and will be fluent in both English and Kiswahili.

(b) For projects that will involve community members (e.g., peer researchers) in the collection and/or analysis of data, please describe their status within the research team (e.g., are they considered employees, volunteers or participants?) and what kind of training they will receive?

All research assistants will be considered employees and will be paid a standard per diem or hourly rate, depending on the services provided. We will consult with local researchers to determine appropriate rates of pay. Some research team members (i.e. project facilitators, interpreters, translators) may already be employed by World Vision for the purpose of the larger CIDA-funded SUSTAIN project; these individuals will either be paid in kind, or will be given an extra stipend if they are required to work outside of their regular hours.

All research assistants involved in data collection and management will be asked to obtain an online National Institutes of Health Research Ethics Training certificate, and will be required to sign a confidentiality agreement prior to their participation. KH will also train them in relevant research methodologies and data analysis techniques.

15. RECRUITMENT OF PARTICIPANTS

- Where there is recruitment, please describe how, by whom, and from where the participants will be recruited
- Where participant observation is to be used, please explain the form of insertion of the researcher into the research setting (e.g., living in a community, visiting on a bi-weekly basis, attending organized functions)
- If relevant, describe any translation of recruitment materials, how this will occur and whether or not those people responsible for recruitment will speak the language of the participants.
- Attach a copy of all posters, advertisements, flyers, letters, e-mail text, or telephone scripts to be used for recruitment.

Recruitment Procedures for CHWs

a) Currently, a total of 76 CHWs work in the mHealth intervention area. World Vision will randomly allocate the mobile phone intervention to 32 of these CHWs (16 in each of the two catchment areas – Mtinko and Kisiriri). A research team member will contact each of these CHWs at the end of the mHealth training period. Following the training, a member of the research team will describe the study to the 32 CHWs as a group. Those who would like to participate will be given a written consent form. A research team member will read through this form aloud, and then answer any questions the CHWs might have.

b) We will recruit an additional 32 CHWs for the control group, following the same distribution across the two catchment areas. We will follow the same cluster-randomized design as the intervention, and thus will randomize by village. Initial contact with potential control group CHWs will be made during the Ministry of Health Training Session on MNCH in Singida town, which will be organized and facilitated by World Vision. Those CHWs who work in the randomly selected villages will be asked to remain after the training session to discuss the study with the research team. At the end of the training session, a member of the research team will follow the same recruitment/consenting process as in a) above. For those who happen to be absent at the time of recruitment/enrolment (due to illness, family emergency, or if they were previously trained, for example), a member of the research team will contact them by phone. The research assistant will describe the study over the phone, and if the CHW is interested in the study, they will be invited to participate in an interview. In this situation, written informed consent will be obtained when the CHWs arrives for their interview.

To ensure that consent is indeed informed, all recruitment and enrolment materials will be translated into Swahili. We will consult with Tanzanian colleagues at WV to ensure these materials are written in appropriate language given the education levels of CHWs. We do not anticipate any difficulties with comprehension of the consent process, given local educational requirements for CHW training programs.

As outlined in the consent form, participants will reserve the right to withdraw from the study at any time.

Recruitment procedures for women (CHW clients):

A) Recruitment of women for first round of Focus Group Discussions:

Four experimental and 4 control villages will be selected purposively for focus groups with pregnant women. We will review the paper-based monthly reports for control group CHWs, and the CommCare database for experimental group CHWs in order to select the villages in which CHWs are most active (measured by the number of new pregnant women each CHW registered in the last

month). Women fitting study inclusion criteria will be selected from CHWs' health registration records. We aim to invite 6-8 women per FGD, as per best practice guidelines.

Recruitment of women for focus group discussions (FGDs) will occur using the steps below. These steps are designed to both minimize selection bias, create a framework for monitoring any particular bias in FGD attendance, and should minimize any potential for undue influence:

1. A World Vision staff member who is already working closely with community health workers (CHWs) will request a complete list of all currently pregnant clients from CHWs working in target villages.

2. If the total number of women is less than or equal to 8, then the CHW will invite all of them to participate in the FGD.

3. If the number of women is greater than 8, then the research team will choose at random which women are to be invited (not CHWs themselves).

Thus, there should be no bias due to CHW influence on selection of women to be invited.

There may, however, be bias on which women are already clients, but since the study is designed to monitor people served by the intervention, this particular bias is not relevant to the sampling protocol. In other words, if CHWs are deliberately not taking certain women on as clients, this is a programmatic issue we cannot control.

CHWs will facilitate the logistics to bring all women selected for invitation to the focus groups. There is no feasible alternative to this approach because we are dealing with a very rural, scattered area, and few target participant women have phones. Further, the CHWs are the only local community resource persons with full knowledge of the target samples relevant. While CHWs will help to facilitate the gathering of potential participants, the women will still have the opportunity to decide whether to participate (or not) after CHWs leave. In other words, CHWs will *invite* women to participate, but will not be the ones to enroll them. Neither CHWs nor World Vision staff will be present during FGDs. Discussions will be held in a private area, away from both the clinic and World Vision office.

Because there will be full monitoring of women's participation, the research team will have an indicator for any CHWs where attendance and participation rates of women volunteering is noticeably lower. If that happens, we will be able to assess this bias and potentially exclude data if necessary.

Village government officials (executive officer or chairperson) will be advised that the study will take place in their jurisdiction, by letter or by phone. This is in keeping with local political protocol. These parties will not be involved in the recruitment process so as to avoid any potential for undue influence.

B) Programmatic data is routinely collected on paper-based registers by CHWs as part of regular World Vision programming. CHWs will maintain a list of newly pregnant women in their communities throughout the intervention period. Those women meeting inclusion criteria will be approached for recruitment (described in (B) below). Permission to conduct secondary analysis on pregnant women's medical information (programmatic data – collected via CHW registers) will be obtained from each woman directly, by members of the research team, independent of CHWs. Women will be asked to indicate on the consent form whether they agree to participate in this component of the

research. The consent form will be presented prior to the household survey at the end of the intervention period.

The data received from World Vision by the research team will be completely de-identified. We will not extract this programmatic data from CHWs' registers until the end of the intervention period and we will only use aggregate data in the analysis. We will only perform secondary data analysis for those women who provided consent.

C) Recruitment of women for household surveys:

Since programmatic data collected by CHWs is subject to bias and is often incomplete and/or inaccurate, we will recruit a sub-sample of women at the *end* of the intervention period to participate in one household survey. The household survey will also give us the opportunity to collect additional data not routinely collected by CHWs (e.g. perceived quality of care, socioeconomic indicators, etc.). Research team members will conduct the survey independently of CHWs.

Prior to the household survey, we will obtain from CHWs complete lists of their clients based on their home visit registers.

From September – May 2014, we will ask CHWs to keep a list of newly pregnant women in their villages as well as their contact information. We will ask CHWs to collect mobile phone numbers for their clients. For women who do not have their own mobile phone, CHWs will ask if another household member or neighbor has one.

At the end of the intervention period, a member of the research team will collect the list of mothers' names and contact information. The identifying information will not be linked to any research data – the list of names will be used to generate a unique study ID for each participant.

The research team will organize the list of client names according to village and geographic area. For those women who provided telephone numbers, a member of the research team will phone them first to ask if they would be available to meet briefly to discuss a research study. For women who did not provide a contact phone number, we will ask CHWs to help us locate participants and make household visits. CHWs will be asked to inform selected women of the date and purpose of the visit ahead of time.

Sampling Protocol for household surveys:

a. Obtain complete lists of clients meeting study inclusion criteria from each CHW.
b. If a CHW has more than 12 eligible women on their list, prioritize those with longer exposure time (i.e. visited by a CHW prior to 6 months gestation).
c. Randomly select from remaining client names until N = 12 for each CHW.
d. If a CHW has less than 10 eligible clients, recruit/invite all of them to participate. In these cases, to reach our target of N = 20 for each village, select additional clients from the second CHW's list to make up the difference.
e. In villages where CHWs have more than 20 eligible clients, randomly select up to 24 names (with a balance between CHWs wherever possible).
f. In cases where selected women are not present (e.g. traveling) or unreachable the day of the survey, replace this client with another randomly selected name, if possible.

If a CHW accompanies research team members to the women's homes, they will not be present for the actual survey. A research team member will conduct all surveys in private.

Arrangements will be made with these mothers to either meet them at their local dispensary or to meet at their homes, depending on their preference. If women are planning a trip to the dispensary, then a research team member will meet with them at the facility, prior to or following their visit with health personnel. In order to increase efficiency and minimize cost, research team members will create a strategic schedule for visiting and recruiting the women in clusters.

Both CHWs and pregnant women will always be recruited by a research team member who speaks Kiswahili.

Recruitment procedures for health facility staff:

At the midpoint of the study (estimated November 2013), we will recruit 1 staff member from each health facility for interviews (N = 14). Because World Vision works closely with these dispensaries, they will be able to facilitate contact with clinic staff. A Swahili-speaking member of the research team will contact these individuals by mobile phone and ask if they would be interested in participating in a semi-structured interview. If agreeable, we will arrange to visit the dispensary and conduct the interview at a time that is most convenient to the clinic staff (i.e. just before or just after their shift). Written consent will be obtained from each participant.

16. COMPENSATION

Please see U of T's Compensation and Reimbursement Guidelines.

(a) Will participants receive compensation for participation?

Financial	Yes ☐	No ☒
In-kind	Yes ☒	No ☐
Other	Yes ☐	No ☒

(b) If **Yes**, please provide details and justification for the amount or the value of the compensation offered.

For the qualitative components (interviews and focus groups) with CHWs, reimbursement for travel to World Vision offices will be provided but there will be no extra stipend. Depending on the time requirement, lunch and/or light refreshments may also be provided. This is established best practice in this type of research and colleagues at World Vision Tanzania have confirmed that this is a culturally appropriate compensation method in the anticipated study areas. In addition, we do not CHWs not involved in the study to misinterpret the purpose of the payments; this could lead to perceptions of inequity, which would have a negative impact on World Vision's programming

There will be no need to reimburse mothers for interviews and focus groups because research team members will travel to conduct this research within women's communities. A small gift of fruit or baby socks will be provided to mothers as a token of appreciation for their time and information.

CHWs will assist with logistics of locating women participants and helping research team members find participants' households. Our colleagues at World Vision have informed us that when CHWs are asked to assist with household surveys, it is standard local practice to give them a small monetary stipend. We have been advised to provide each CHW with 5000 TZS (approx. $3). Therefore, CHWs will not be compensated for their participation in the research study, but they will be paid a small stipend for helping with logistics during the household survey with clients.

(c) If **No**, please explain why compensation is not possible or appropriate.

No cash incentives will be given to participants to eliminate one source of undue influence on individuals to participate, avoid raising inappropriate expectations of potential additional payments and reduce any perceptions of inequity. This approach also improves likelihood that participants will exercise their right to withdraw without foregoing a financial incentive.

(d) Where there is a withdrawal clause in the research procedure, if participants choose to withdraw, how will compensation be affected?

Compensation will not be affected if participants choose to withdraw.

SECTION C –DESCRIPTION OF THE RISKS AND BENEFITS OF THE PROPOSED RESEARCH

17. POSSIBLE RISKS

(a) Please indicate all potential risks to participants as individuals or as members of a community that may arise from this research:

(i) Physical risks (e.g., any bodily contact or administration of any substance): Yes ☐ No ☒

(ii) Psychological/emotional risks (e.g., feeling uncomfortable, embarrassed, or upset): Yes ☒ No ☐

(iii) Social risks (e.g., loss of status, privacy and/or reputation): Yes ☐ No ☒

(iv) Legal risks (e.g., apprehension or arrest, subpoena): Yes ☐ No ☒

(b) Please briefly describe each of the risks noted above and outline the steps that will be taken to manage and/or minimize them.

This study imposes very little risk to either CHW or their clients. The nature of data collection is non-invasive and poses no physical or biological risks to participants. A trained research team member will be trained and required to protect confidentiality of participants during the collection of quantitative medical information from women's health registers. All information will be de-identified by removing this information prior to entry into the study database as one essential step to establish and maintain anonymity.

Qualitative interviews and focus groups will impose minimal risk on participants. CHWs will be asked questions regarding their work including job satisfaction, perceived barriers and facilitators, and attitudes towards mHealth. These topics are not expected to cause any anxiety or stress among participants. Nonetheless, it will be stressed that participation is completely voluntary and CHWs do not have to answer any questions that they feel uncomfortable answering.

In interviews and focus groups, Pregnant women/mothers will be asked questions regarding their opinions of care provided by CHWs, home birth vs. facility birth, attitudes towards biomedical vs. traditional birth attendants, barriers to accessing maternal health services, and maternal healthcare needs. While these topics are not overly invasive, there is a slight risk of participants experiencing

feelings of anxiety, stress or embarrassment regarding these topics. To minimize this risk, all subjects will be informed of the nature of the study, and the topics of interest prior to participation, and we will seek written consent from each participant. It will be stressed that participation is completely voluntary. When subjects feel uncomfortable answering a particular question, they will not be pressured by the PI or other data collectors to continue the conversation. Subjects will have the right to withdraw from the study at any time.

We will ensure that participants do not feel pressured to participate by emphasizing that the study is completely voluntary and that their decision to participate (or not) will have no impact on a) their relationship with World Vision or the Ministry of Health; or b) any healthcare they receive.

There is also a risk that participants may feel pressured to provide information they believe is desirable by the data collector during interviews, surveys and focus groups. To ensure that participants answer honestly, the interviewer/data collector will explain that their answers will be kept completely confidential, and that no identifying information will ever be linked to the information they provide.

There is always some risk in focus group discussions that someone will share something that they later regret having shared, leading to a feeling of embarrassment or of a loss of privacy. We will minimize this risk by explaining at the start of the focus groups that we cannot guarantee confidentiality because we will be discussing information as a group. Therefore, if participants feel uncomfortable sharing any of their statements with others in or outside the group, then they should not share them during focus group meetings. In addition, we will ask that each participant respect the confidence of others during focus groups by not sharing details of the conversation with anyone outside of these discussions.

Focus Group facilitators will openly discuss with participants the importance of our collective responsibility to be sensitive and confidential with respect to comments, questions and perceptions of others. We will be particularly sensitive to the emotions and feelings of participants so that the conversation may be moderated appropriately.

During both the preparatory phase and the research phase itself, advice will be sought from local researchers to determine which techniques and approaches will be most appropriate for facilitating confidential participation in the Tanzanian context. We realize the need for facilitators to be alert and mindful of sensitive topics that may emerge during focus group discussions and find ways to either change the topic or redirect a question if the topic appears to cause anxiety among participants. Probing is a powerful tool for eliciting information and also for shifting the topic of discussion in focus group research. This is certainly a tool we will use during focus groups to minimize any emotional risk posed to participants.

We will conduct interviews and focus groups in a private space, removed from the community whenever possible. We will make every effort to ensure that no family members, neighbours or World Vision staff are present or in the vicinity. If possible, we will hold interviews with CHWs at the Aqua Vitae Hotel in Singida, where CHWs frequently receive trainings and participate in workshops. This location is ideal for interviewing because the space if private, and CHWs will be removed from both their community and World Vision staff. Research team staff will be trained to judge when to stop/proceed with an interview or FGD, and to manage other people who may be in the vicinity. In cases where others *are* present (for example, if a CHW insists on having a friend/family member or colleague present) we will make note of this and analyze the data accordingly.

During follow-up focus groups with CHWs and women clients, it will be emphasized that we cannot guarantee confidentiality therefore participants should share information accordingly. Focus groups

will also be conducted in a private space (at the social training centre, if possible); World Vision staff will not be present. Further, all transcripts in both Swahili and English will not include any identifying information. Only participant ID codes will be used. Lastly, transcripts and recordings will be stored on an encrypted database to which only the PIs will have access.

Lastly, any results discussed in focus groups or interviews will be aggregated data and will not be attached to any personally identifying information. This will be explicitly communicated to potential participants.

Additional risk:

There is a risk that CHWs may engage in unprofessional or inaccurate practices with clients, however our protocol is not designed to capture these data. If such practices occur, it is not a direct result of the evaluation research, as these are ongoing concerns to be addressed by World Vision program staff. As external evaluators of World Vision's mHealth intervention, we have not been asked to assess their interactions with clients directly. World Vision monitors this as part of their program management strategy, however we will not be able to link any of this programmatic data to specific CHWs.

Although this is outside the scope of our research objectives, it is possible that information about unprofessional practices by CHWs may emerge during discussions with women clients in our study. If any concerns regarding unethical or unprofessional practices are brought to our attention, we will immediately report this information to World Vision program officers so that they may follow up appropriately.

18. POSSIBLE BENEFITS

- Describe any potential direct benefits to participants from their involvement in the project
- Describe any potential direct benefits to the community (e.g., capacity building)
- Comment on the potential benefits to the scientific/scholarly community or society that would justify involvement of participants in this study

Results of this research will be used to develop recommendations on ways to strengthen ongoing and future MNCH programming within Tanzania. Lessons learned regarding the use of mHealth technology and the positioning of CHWs will be invaluable to policy makers and local stakeholders. If the mHealth application is shown to be effective, efforts to scale out the intervention will benefit individuals beyond those enrolled in the study. Ultimately, results of this research may contribute to the strengthening of Tanzania's primary health care system, and the enablement of more equitable access to maternal health services for Tanzanian women.

CHWs may benefit directly from this study because they will be given the opportunity to share their experiences and concerns about their job with researchers. In our experience, this opportunity tends to result in positive experiences for health workers because it validates their work, has an empowering effect, and allows them to participate in the formation of recommendations for future programming.

The increasing penetration of mobile phones in Tanzania presents an unprecedented opportunity to leverage mHealth technologies for maternal and child health. Consequently, there has recently been a rapid increase in mHealth pilot studies throughout Tanzania. While mHealth is thought to be a promising tool, rigorous scientific studies evaluating the reach, effectiveness, and equity of these

interventions are currently lacking. There is need to build an evidence base illustrating the impact of mHealth interventions; the proposed evaluation study aims to fill this gap.

SECTION D – INFORMED CONSENT

19. CONSENT PROCESS

(a) Describe the process that will be used to obtain informed consent and explain how it will be recorded. Please note that it is the quality of the consent, not the form that is important. The goal is to ensure that potential participants understand to what they are consenting.

(b) If the research involves extraction or collection of personally identifiable information from or about a research participant, please describe how consent from the individuals or authorization from the data custodian (e.g., medical records department, district school board) will be obtained.

This study will require several separate consenting processes:

1) CHWs:

Experimental arm: We will seek consent from all CHWs in the mHealth experimental group (N = 32). These CHWs will initially be approached during the mHealth training period. At the end of the training period, a member of the research team will describe the study to the 30 CHWs as a group. Those who would like to participate will be given a written consent form. A research team member will read through this form aloud, and then answer any questions the CHWs might have. It will be emphasized that participation in the evaluation study is completely voluntary, and their decision to participate will not influence their jobs or any relationship they currently have with World Vision. A brief, self-administered baseline survey will be completed by those CHWs who consent to enroll in the study. Participants will also be informed that they will be contacted about halfway through the intervention period to participate in a semi-structured interview, and then could potentially be selected to participate in an end line focus group discussion. Participants will be required to sign a separate consent form each time they participate in a research activity.

Control arm: We will recruit an additional 32 CHWs for the control group, following the same distribution across catchment areas. We will follow the same cluster-randomized design as the intervention, and thus will randomize by village. Initial contact with potential control group CHWs will be made during the Ministry of Health Training session on Maternal Newborn and Child Health. Those CHWs who work in the randomly selected villages will be asked to remain after the training session to discuss the study. A member of the research team will describe the study to the CHWs as a group, and explain that participation is completely voluntary. CHWs who are interested in participating will be given a written consent form. A research assistant will read through the form aloud, and then answer any questions the CHWs might have. At this time, a self-administered baseline survey will be completed by those CHWs who consent to enroll in the study. CHWs will also be asked to participate in interviews and focus groups at a later date.

As described on page 15 above, those CHWs who are absent on the day of enrolment will complete a written informed consent form (and the baseline survey) on the same day as their interview.

2a) CHWs' clients – qualitative component

A sub-sample of ~24 female clients will be randomly selected from each study arm (total N = 48) to participate in 8 focus group discussions (FGDs). As described above, potential participants will be

selected from CHWs' records/lists, then contacted by phone. Women who express interest in participating will be asked to attend a focus group discussion at a private location in their village or a village nearby. Arrangements for focus groups will be made with the help of World Vision staff and village leaders (members of village government). Before beginning the focus group, the facilitator will read through the consent form aloud, and then answer any questions the women might have. Participants will be encouraged to ask questions before signing consent forms.

Participants will be asked to indicate whether they would be interested in participating in a follow-up focus group near the end of the intervention period.

2b) CHWs' clients (women) – household surveys

As described in section 15 (above), we will utilize a combination of CHWs' client records and clinic records as a pregnancy surveillance system. To obtain consent from women who were pregnant during the intervention period of interest (September – November 2013), a member of the research team will contact potential participants via mobile phone (if available) ahead of time to arrange a meeting time for the household survey. Arrangements will be made with women to meet them at either their local dispensary or to meet at their homes, depending on participant preference. If women are planning a trip to the dispensary for an ANC appointment, then a research team member will meet with them at the facility, prior to or following their visit with health personnel. For those who are unreachable by phone, CHWs will be asked to accompany members of the research team to the women's villages, where consent will be obtained at women's homes.

During meetings with potential participants, research assistants will be provided with a brief script describing the study, which they will read aloud to each woman. This will include a description of potential risks and benefits of the research. This script will be pilot-tested to ensure that the language used is culturally appropriate and comprehensible. Women will be encouraged to ask questions if anything is unclear or if they have specific concerns about the research process. It will be emphasized that all data collected will be de-identified, and their decision to participate in the study (or not) will in no way impact on the medical care they receive from CHWs or other health personnel. If a woman agrees to participate, she will be asked to provide her signature or a thumbprint (if unable to write) on the consent form. Women who agree to participate will be asked to complete a semi-structured survey at this time.

2c) CHWs' clients (women) – programmatic data

As described above, permission to use women's medical information from CHW registers (programmatic data) will be obtained from each woman directly by members of the research team, independent of CHWs. On the consent form, there are two separate sections requiring signatures: 1) participation in household survey; and 2) permission for use of CHW register data for secondary analysis. Thus, women will have the option of participating in both components of the study, or they may choose to participate only in one. Data collectors will explicitly inform women that involvement in the research is completely voluntary, and separate from health care, and that their decision to participate will have no impact on the care received from CHWs. The data received from World Vision by the research team will be completely de-identified. We will only use aggregate data in this part of the analysis. This will also be emphasized during the consent process.

The data received from World Vision by the research team will be completely de-identified. We will only use aggregate data in this part of the analysis.

The contact number of a Kiswahili-speaking research team member will be included on the consent form, in case participants have questions after the survey.

Because we will be conducting research within the context of an existing community-based health program, World Vision is the custodian of the programmatic data. By contracting U of T to conduct an evaluation of the mHealth intervention, they have authorized the use of this data for research purposes.

All research participants will be recruited and consented by a research team member who speaks Kiswahili fluently.

20 CONSENT DOCUMENTS

(a) **Attach a copy of the Information Letter/Consent Form.**
For details about the required elements in the information letter and consent form, please refer to our informed consent guide (**http://www.research.utoronto.ca/wp-content/uploads/2010/01/GUIDE-FOR-INFORMED-CONSENT-April-2010.pdf**)

Additional documentation regarding consent should be provided such as:

a. **screening materials introductory letters, letters of administrative consent or authorization**

(b) If any of the information collected in the screening process - prior to full informed consent to participate in the study - is to be retained from those who are later excluded or refuse to participate in the study, please state how potential participants will be informed of this course of action and whether they will have the right to refuse to allow this information to be kept.

Not applicable.

21. COMMUNITY AND/OR ORGANIZATIONAL CONSENT, OR CONSENT BY AN AUTHORIZED PARTY

(a) If the research is taking place within a community or an organization which requires that formal consent be sought prior to the involvement of individual participants, describe how consent will be obtained and attach any relevant documentation. If consent will not be sought, please provide a justification and describe any alternative forms of consultation that may take place.

Prior to developing a detailed protocol, we discussed project plans with the local Ministry of Health authority (regional medical officer - RMO). The RMO was enthusiastic about the project and gave verbal permission. We have since obtained an official permission letter from the RMO, which was submitted along with a complete research protocol to Tanzania's National Institute for Medical Research (NIMR), the national research ethics body. NIMR approved the study protocol on July 24th, 2014.

(b) If any or all of the participants are children and/or others who are not competent to consent, describe the process by which capacity/competency will be assessed, and the proposed alternate source of consent.

i) Submit a copy of the permission/information letter to be provided to the person(s) providing the alternative consent

ii) Describe the assent process for participants and attach the assent letter.

Children will not participate in this research and women's age will be confirmed as at least 16 by CHWs. Very young infants (less than 6 months) may be referred to in responses by mothers who have given consent to participate. It is intended that only women competent to give consent will be recruited into the study. Resource limitations will mean that the only criteria for assessment of competency will be the unsystematic professional opinion of field staff.<br><br>In cases where the mothers have limited functional literacy, the recruiting team member will sign the consent form to confirm that the statements have been read. The participant's verbal consent will also be digitally recorded prior to the focus group/interview. It is anticipated everyone will be able to provide a signature.

22. DEBRIEFING and DISSEMINATION

(a) If deception or intentional non-disclosure will be used in the study, provide justification. Please consult the Guidelines for the Use of Deception and Debriefing in Research

Not applicable.

(b) Please provide a copy of the written debriefing form, if applicable.

Not applicable.

(c) If participants and/or communities will be given the option of withdrawing their data following the debriefing, please describe this process.

Not applicable.

(d) Please describe what information/feedback will be provided to participants and/or communities after their participation in the project is complete (e.g., report, poster presentation, pamphlet, etc.) and note how participants will be able to access this information.

Knowledge Transfer: Following data analysis, We will develop a report summarizing the major findings and recommendations for both WV and Tanzania MoH. We will organize a symposium where we will present these results to key local stakeholders including politicians, health care professionals and local health ministers.<br><br>KH will also return to Singida region to hold debriefing meetings at ADP offices in each study area. CHWs enrolled in the study will be invited to attend these meetings. She will disseminate the information orally, answer any questions the CHWs might have, and solicit and record feedback on the study and findings, and will discuss future research/programmatic opportunities with the CHWs.<br><br>We will also disseminate results among the research community through workshops, conference presentations, and peer-reviewed journals.

23. PARTICIPANT WITHDRAWAL

(a) Where applicable, please describe how participants will be informed of their right to withdraw from the project and outline the procedures that will be followed to allow them to exercise this right.

During the consent process, participants in both target groups will be informed of their right to withdraw from the study at any point prior to the data analysis phase (**July 2014**). We will include the contact information (including a phone number) for a Kiswahili-speaking member of the research team in the consent letter. In order to withdraw from the study, participants will contact this research team member.

It is not feasible for participants to withdraw information they share in focus group discussions, as this is shared in a group setting. However, it will be stressed prior to the beginning of the focus group discussion that participants should not feel obligated to provide any information that they are uncomfortable sharing.

(b) Indicate what will be done with the participant's data and any consequences which withdrawal may have on the participant.

The decision to withdraw from the study will have no consequences on the participant. It will be stressed during the consenting process that research activities are completely separate from World Vision's community health programming, thus healthcare received through this program will not be influenced by their decision to withdraw from the study.

Participants will be informed during the consent process that the data they provide prior to the withdrawl date will still be retained and included in analysis. However, should participants specifically request to withdraw some portion of the information or all of the information provided, we will completely destroy that information and no portion of the withdrawn information will be used in further stages of the research.

(c) If participants will not have the right to withdraw from the project at all, or beyond a certain point, please explain. Ensure this information is included in the consent process and consent form.

Not applicable.

SECTION E – CONFIDENTIALITY AND PRIVACY

24. CONFIDENTIALITY

Data security measures must be consistent with UT's *Data Security Standards for Personally Identifiable and Other Confidential Data in Research*. All identifiable electronic data that is being kept outside of a secure server environment must be encrypted, consistent with the standards described at: *http://www.utoronto.ca/security/UTORprotect/encryption_guidelines.htm:*

(a) Will the data be treated as confidential? Yes ☒ No ☐

(b) Describe the procedures to be used to protect the confidentiality of participants or informants, where applicable

To ensure anonymity of the participants, unique study ID codes will be used to identify participants and groups in all stages of this research. Participants' names and other personal information will never be linked to their responses or personal data.

All identifying information will be modified in case studies cited in written reports and only aggregated data will be disseminated during focus groups or community dissemination meetings.

To protect confidentiality, data collectors will discuss issues of confidentiality with all participants and any research assistants will be asked to sign a confidentiality agreement. Additionally, all codes, notes, files, and recordings will be stored on one computer. This computer will be encrypyted and all research files will be password-protected. These files will only be accessible by KH and one other research assistant.

Hard copies of consent forms, written notes, and any other study materials will be locked in a filing cabinet in a secured World Vision office in Singida. Only KH will hold the key to this cabinet.

Survey data collected on tablets will be de-identified; only anonymous participant ID codes will be used. At the end of each research day, all tablets will be synced over wifi to a secure password-protected Google server then manually transferred to an encrypted/password-protected data file. Once the data is transferred to the server, it will be cleared from the tablet's storage folder.

(c) Describe any limitations to protecting the confidentiality of participants whether due to the law, the methods used, or other reasons (e.g., a duty to report)

Anonymity and confidentiality are impossible to guarantee during focus groups. Therefore, to minimize threats to confidentiality as much as possible, at the start of each focus group, we will remind participants that we cannot guarantee confidentiality because we will be discussing information as a group. Therefore, if anyone feels uncomfortable with any of their statements being shared with others in or outside the group, we will suggest that they do not share them during the focus group. We will also ask the participants to please respect the confidence of others by not sharing the information discussed with anyone outside of the group.

Any results from the interviews will be aggregate data and will not be linked to any personal identifiers.

25. DATA SECURITY, RETENTION AND ACCESS

(a) Describe how data (including written records, video/audio recordings, artifacts and questionnaires) will be protected during the conduct of the research and dissemination of results.

All codes, notes, files, and recordings will be stored on one computer will be stored in separate, password-protected files to which only KH will have access, and all written notes and files will be locked in a filing cabinet in the World Vision office, to which only KH will hold the key. Any research files stored on KH's personal computer will be password-protected. We will purchase data encryption software and encrypt all data stored outside a secured server.

Data security during transport to Canada: All data files will be encrypted prior to departure from Tanzania. All data will be stored on KH's personal laptop, which she will carry at all times. KH will travel from Singida town to the airport in a World Vision vehicle, thus the risk of stolen property while en route is minimal. At the airport and on the plane, KH will have her laptop in her carry-on luggage; it will never be left unattended.

(b) Explain how long data will be retained. (If applicable, referring to the standard data retention practice for your discipline) Provide details of their final disposal or storage. Provide a justification if

you intend to store your data for an indefinite length of time. If the data may have archival value, discuss how participants will be informed of this possibility during the consent process.

All hard copies of consent forms and digital recordings will be destroyed upon the publication of results from this study. Since the data gathered during this research will inform future interventions involving mHealth and CHWs, we will keep the research notes and files for 7 years following the publication of research results, after which time they will be completely destroyed. All identifiers will have been stripped from these notes and files during my research, and the codes will be stored in a separate, password-protected file on my computer, to which only study select research team members will have access during and after the field research period.

(c) If participant anonymity or confidentiality is not appropriate to this research project, please explain.

Not applicable.

(d) If data will be shared with other researchers or users, please describe how and where the data will be stored and any restrictions that will be made regarding access.

Not applicable.

SECTION F – LEVEL OF RISK AND REVIEW TYPE

See the *Instructions for Ethics Review Protocol Submission Form* for detailed information about the Risk Matrix.

26. RISK MATRIX: REVIEW TYPE BY GROUP VULNERABILITY and RESEARCH RISK

(a) Indicate the Risk Level for this project by checking the intersecting box

	Research Risk		
**Group Vulnerability**	**Low**	**Medium**	**High**
**Low**	**1** ☐	**1** ☐	**2** ☐
**Medium**	**1** ☐	**2** ☒	**3** ☐
**High**	**2** ☐	**3** ☐	**3** ☐

(b) Explain/justify the level of research risk and group vulnerability reported above:

We feel the research risk is relatively low, given that we will employ non-invasive research techniques (observations, brief surveys, interviews, focus groups, and data extraction from patient health records). In addition, participation is voluntary, and respondents will be given the option to withdraw from the study at any time. Data from participants' health records will be de-identified and every effort to maintain anonymity will be made throughout the entire research process. There is always a chance that information provided will be linked back to specific individuals, however we have strong confidentiality, data protection, and data management procedures in place to mitigate this potential risk. We feel that this study design will protect participants by a) ensuring that feedback regarding CHWs' quality of care is not going to influence their job; and b) ensuring that information provided by female clients will not result in differential treatment provided by CHWs. While we feel

the study poses low risk, this boundary can be blurry. Therefore, we are erring on the side of caution and classifying the research risk as medium.

We believe the group vulnerability level is medium because CHWs' clients are all pregnant women and many will have limited education, household income and access to water, sanitation, food and primary health care. Although pregnant women are typically considered a vulnerable population, the topics that will be discussed with them will not normally be controversial. In the event that an uncomfortable topic arises during interviews and focus groups, facilitators/interviewers will monitor the body language and tone of participants and will change the subject if necessary. We will also employ a referral system whereby interviewers will refer participants describing unmanaged illness to local community health facilities. If any research team members come across women or children requiring medical attention, they will be referred to the local health facility to be seen by a trained clinician.

(Please note that the final determination of Review Type and level of monitoring will be made by the reviewing University of Toronto REB)

Based on the level of risk, these are the types of review that a protocol may receive:

Risk level = 1: Delegated Review;   Risk level = 2 or 3: Full Board Review

For both delegated and full reviews (SSH&E, HS, or HIV), please submit one electronic copy of your protocol and all appendices (e.g., recruitment, information/consent and debriefing materials, and study instruments) as a **single** Word document or a pdf. *Do not submit your entire research proposal.* Please ensure that the electronic signatures are in place and e-mail to **new.ethics.protocols@utoronto.ca**

The deadline for delegated review (SSH&E or HS) is EVERY Monday, or first business day of the week, by 4 pm. Information about full REB meeting and submission due dates are posted on our website (SSH&E, HS or HIV).

HIV REB reviews all protocols at full board level but applies proportionate review based on the level of risk.

All other submissions (e.g., amendments, adverse events, and continuing review submissions) should be sent to ethics.review@utoronto.ca
