# Supplementary material for: Impact of smartphone-assisted prenatal home visits on women’s use of facility delivery: Results from a cluster-randomized trial in rural Tanzania
Source: PLoS One. 2018 Jun 18;13(6):e0199400. doi: 10.1371/journal.pone.0199400 (PMC6005474; doi:10.1371/journal.pone.0199400)
Supplement: S1 Questionnaire — (PDF) [file pone.0199400.s004.pdf]

CONFIDENTIAL: For research purposes only

UNIVERSITY OF TORONTO

Household Survey for Women (CHW clients)

****note: this survey has been reformatted and programmed to be administered on tablet devices****

ADMINISTRATIVE INFORMATION:

Initials of research assistant: ______________	Village ID: -----------------
Date of survey: -------- / ------- / ---------- (day / month / year)	Participant ID: -----------------

SOCIODEMOGRAPHIC INFORMATION:		
During the survey, please ask for help if you do not understand a question.<br>First I will ask some general questions about you and your household.		
1. What is your date of birth?	-------/-------/--------<br>day / month / year	
2. What is the highest level of schooling that you've completed?	None ------------------------------	00
	Non-formal -----------------------	1
	Primary 1-4 -----------------------	2
	Primary 1-7 -----------------------	3
	Secondary ------------------------	4
	Advanced secondary ------------	5
	College/University --------------	99
3. What is the main source of drinking water for the household?	Borehole or Piped water --------	1
	Protected well/spring -----------	2
	Rainwater collection ------------	3
	Bottled water ---------------------	4

	Unprotected dug well/spring ---<br>Pond, river, stream --------------<br>Don't know ----------------------<br>Other _____________________<br>(specify)	5<br>6<br>88<br>99
4. What type of toilet facilities does your household use?	VIP latrine or Flush toilet ------<br>Traditional or open pit latrine -<br>Bucket ---------------------------<br>No facilities, use bush or field -<br>Other ___________________<br>(specify)	1<br>2<br>3<br>4<br>99
5. What is your religion?	Christian -------------------------<br>Muslim --------------------------<br>None -----------------------------<br>Other _____________________<br>(specify)	1<br>2<br>88<br>99
6. What is the main method of transportation you use to travel to the health facility?	By foot ---------------------------<br>Bus -------------------------------<br>Taxi ------------------------------<br>Bicycle ---------------------------<br>Motorbike -----------------------<br>Other _____________________<br>(specify)	1<br>2<br>3<br>4<br>5<br>99
7. How long does it take you to travel to clinic by this method?	_______ hours<br>_______ minutes	

HEALTH CARE DELIVERY BY COMMUNITY HEALTH WORKERS

I would like to ask your opinion about community health workers' care and services. I will read several statements to you. After the statement is read, please tell me whether you "strongly agree", "agree", "disagree", or "strongly disagree".

	Strongly Agree	Agree	Disagree	Strongly Disagree	Unsure	
8. Community health workers know a lot about the health of mothers.	3	2	1	0	99	
9. Community health workers know a lot about child health.	3	2	1	0	99	
10. Community health workers are not very good at diagnosing child illnesses.	0	1	2	3	99	
11. I trust the health advice given by community health workers	3	2	1	0	99	
12. Community health workers are good at monitoring their clients over time.	3	2	1	0	99	
13. Community health workers have a lot of useful skills.	3	2	1	0	99	
14. Community health workers do not provide good solutions to my family's health problems.	0	1	2	3	99	
15. Community health workers have the proper tools to take care of mothers and young children.	0	1	2	3	99	

INTERPERSONAL ASPECTS OF CARE

Your answers to the following statements will help me to learn more about your relationship with community health workers in your area. Again, after the statement is read, please tell me whether you "strongly agree", "agree", "disagree", or "strongly disagree".

	Strongly Agree	Agree	Disagree	Strongly Disagree	Unsure	
16. Community health workers are compassionate towards myself and my children.	3	2	1	0	99	
17. Community health workers are disrespectful towards myself and my children.	0	1	2	3	99	
18. Community health workers do not have much time to spend with the women and children they care for.	0	1	2	3	99	
19. I find it easy to talk to community health workers.	3	2	1	0	99	
20. I believe community health workers are honest.	3	2	1	0	99	
21. Overall, I am satisfied with the care provided to my family by community health workers.	3	2	1	0	99	

Now I will ask you some questions about the closest health facility and the staff who work there. Again, after the statement is read, please tell me whether you "strongly agree", "agree", "disagree", or "strongly disagree".

	Strongly Agree	Agree	Disagree	Strongly Disagree	Unsure	
22. Health facility staffs are compassionate towards my children and myself.	3	2	1	0	99	
23. Health facility staffs are disrespectful towards my children and myself.	0	1	2	3	99	
24. Health facility staffs do not have much time to spend with the women and children they care for.	0	1	2	3	99	
25. I find it easy to talk to health facility staff.	3	2	1	0	99	
26. I trust the health/medical advice provided by health facility staff.	3	2	1	0	99	
27. Health facility staff are knowledgeable about maternal and child health.	3	2	1	0	99	
28. Health facility staff are unkind to patients.	0	1	2	3	99	
29. The health facility is always clean and well maintained.	3	2	1	0	99	
30. The health facility has proper medical equipment.	3	2	1	0	99	
31. The health facility often runs out of medication and supplies.	0	1	2	3	99	
32. The health facility is well staffed.	3	2	1	0	99	
33. Line-ups at the health facility are too long.	0	1	2	3	99	
34. Overall, I am satisfied with the care provided to my family by health facility staff.	3	2	1	0	99	

MOST RECENT PREGNANCY & DELIVERY

The next questions are about your most recent pregnancy and delivery.

*__Note to interviewer__:

Where applicable, please confirm the following information by asking to see the participant's clinic card.

35. Did you deliver your most recent baby in a health facility?	YES …………	1
	NO …………..	2
36. If yes, in what type of facility did you deliver this baby?	Dispensary	1
	Hospital	2
	Health Centre	3
37. If yes, was this the nearest health facility to your home?	YES …………	1
	NO …………..	2
38. If you did not give birth in a health facility, please tell us where you delivered.	At home ….........	1
	On the way to facility ……	2
	Other _________________	99

39. If you did not give birth in a health facility, can you tell us why?	I prefer to deliver at home ------------<br>Health facility too far away ----------<br>Health facility too expensive ---------<br>Husband/partner insisted --------------<br>Mother in-law insisted ----------------<br>No transportation ----------------------<br>Other: _______________________<br>(specify)	1<br>2<br>3<br>4<br>5<br>99
40. Did you receive postnatal care (PNC) at a health facility within 2-3 days after childbirth? (*check RCH1 card**)**	YES …………<br>NO …………..	1<br>2
41. How many antenatal care (ANC) clinic visits at a health facility did you attend during your most recent pregnancy?<br>(***check RCH4 clinic card)**	One ………………<br>Two ……………<br>Three ……………<br>Four ……………<br>None ……………..<br>More than Four ………	1<br>2<br>3<br>4<br>0<br>99
42. How many months pregnant were you when you went for the first ANC clinic visit? (***check RCH4 clinic card)**	Number of months	_____
43. When you were pregnant with this child, did you receive or buy any tablets to increase the blood (iron)? (***show tablet***)	YES …………<br>NO …………..	1<br>2
44. I'm not interested in knowing your results, but did you receive HIV testing during this pregnancy?	YES …………<br>NO …………..	1<br>2
45. I'm not interested in knowing your results, but did your partner/husband receive HIV testing during this pregnancy?	YES …………<br>NO …………..	1<br>2
46. Did you take de-worming medication during pregnancy?	YES …………<br>NO …………..	1<br>2
47. Do you have insecticide treated mosquito nets at home?	YES …………<br>NO …………..	1<br>2
48. If yes, who usually sleeps under the net(s)?	My husband only --------------------------<br>Myself only --------------------------------<br>My child(ren) only ------------------------<br>My husband and myself only--------------<br>Myself and my children only	1<br>2<br>3<br>4<br>5

	My husband, myself, and my child(ren)	6
49. During your last pregnancy, how often did you sleep under the net?	Every night --------------------------<br>Most nights --------------------------<br>Some nights -------------------------<br>Never/not at all -----------------------	1<br>2<br>3<br>0
50. Did you sleep under the net last night?	YES …………<br>NO …………..	1<br>2
51. How many doses of malaria prophylaxis (IPT) did you receive during pregnancy? (***check RCH4 clinic card)**	ONE…………..<br>TWO…………<br>NONE ……….	1<br>2<br>0
52. Were you ever referred to a health facility by a CHW during this pregnancy?	YES …………<br>NO …………..	1<br>2
53. If you were referred by a CHW, did you go to the clinic?	YES …………<br>NO …………..	1<br>2
54. If no, why not?		
55. Did your husband/partner accompany you to any ANC appointments?	YES …………<br>NO …………..	1<br>2
56. If yes, how many? ______		
57. Did your husband/partner accompany you during your last delivery?	YES …………<br>NO …………..	1<br>2
58. Did you make a birth plan before delivery?	YES …………<br>NO …………..	1<br>2
59. If yes, who helped you to develop the birth plan?	Health Facility Staff ---------------<br>Community Health Worker ---------<br>TBA -------------------------------<br>Family member -------------------<br>Other: ______________________ (specify)	1<br>2<br>3<br>4<br>99
60. Where did you plan to deliver?	At home ----------------------------<br>Dispensary -------------------------<br>Hospital ----------------------------<br>Relative's home --------------------<br>Other: ________________________ (specify)	1<br>2<br>3<br>4<br>99

61. Was this baby given an oral polio vaccine at birth? (***check RCH1 card)**	YES ………… NO …………..	1 2
62. Was the baby given a BCG vaccination within the first week after birth? (***check RCH1 card)**	YES ………… NO …………..	1 2
63. Have you ever breastfed this baby?	YES ………… NO ………….. **If no, skip to Q?*	1 2
64. How long after birth was the baby put to the breast?	# of hours # of days *If less than 1 hour, record "0" hours. If less than 24 hours, record hours. Otherwise, record days.*	_____ _____
65. Were any other liquids or foods given to the baby during the first week after birth?	YES ………… NO …………..	1 2
	If yes, please list: ______________________________ ______________________________ ______________________________	
66. Are you currently breastfeeding this baby?	YES ………… NO …………..	1 2
67. Now I would like to ask you about liquids or foods (*child's name*) had **yesterday during the day or at night.** Did (*child's name*) drink or eat: **** For each option, circle Y or N***	a. Breast milk…………………………………	Y N
	b. Plain water…………………………………	Y N
	c. Infant formula………………………………	Y N
	d. Any fortified, commercially available infant and young child food (e.g. give local name/brand) ….	Y N
	e. Any (other) porridge or gruel ….. ……………	Y N
68. We'd like to know about your awareness of healthy nutrition practices during pregnancy and breastfeeding. Could you please name any healthy practices that you're aware of? DO NOT READ LIST ALOUD. SELECT ALL THAT ARE MENTIONED.	1. Eat a balanced diet (protein, carbohydrates, fruits, vegetables and fats) 2. Prepare in a hygienic environment 3. Eat three meals a day and snacks in between 4. Eat iron-rich foods (e.g. meat, fish, vegetables) 5. Take iron/folic acid pills during pregnancy and up to 40 days after delivery 6. Use iodized salt 7. Do not overcook vegetables 8. Do not eat things that are not food (e.g. sand/charcoal/ash) 9. Does not know any of the above	

69. We'd like to know about your awareness of danger signs during pregnancy. Could you please name any danger signs that you're aware of?<br>DO NOT READ LIST ALOUD. SELECT ALL THAT ARE MENTIONED.	1. Difficulty breathing<br>2. Fatigue/tiredness<br>3. Vaginal bleeding<br>4. Baby is not moving much or stopped moving completely<br>5. Loss of consciousness or convulsions<br>6. Headache or blurred vision<br>7. Early labour pain before 9 months<br>8. Liquid leaking from vagina (discharge)<br>9. Signs of malaria (feeling cold, fever, vomiting)<br>10. High Blood Pressure<br>11. Swelling of face and arms<br>12. Does not know any of the above
70. We'd like to know about your awareness of danger signs soon after delivery up to 42 days. Could you please name any danger signs that you're aware of?<br>DO NOT READ LIST ALOUD. SELECT ALL THAT ARE MENTIONED.	1. Excessive vaginal bleeding<br>2. Severe abdominal pain or vaginal pain<br>3. High fever or chills<br>4. Fistula<br>5. Loss of consciousness or convulsions (eclampsia)<br>6. Headache or blurred vision<br>7. Swollen breasts or cracked nipples<br>8. Disoriented/abnormal behaviour<br>9. Liquid leaking from vagina (discharge)<br>10. Severe pain in leg muscles<br>11. Does not know any of the above
71. In the past, did you ever experience any difficulties or complications while giving birth (with any of your children)?<br>****If no, skip to next section.***	YES ---------------- 1<br>NO ----------------- 2<br>UNSURE ----------- 3
72. If yes, what complications did you experience?	**SELECT all complications mentioned**:<br>Hemmoraging (exccessive bleeding)<br>Cord around the neck<br>Prolonged labour<br>Eclampsia<br>High Blood Pressure<br>Retained placenta<br>Does not know/remember<br>Other (Please specify) _________________________
73. Who do you prefer to assist you during delivery?	Doctor ---------------------------------- 1<br>Nurse ----------------------------------- 2<br>Medical Officer ------------------------ 3<br>Medical Assistant --------------------- 4<br>Nursing/Medical Student -------------- 5<br>Traditional Birth Attendant ----------- 6<br>Family member ------------------------ 7

	Other _________________ 99<br>(specify)
74. Where is your preferred place of delivery?	Home ---------------------------- 1<br>Dispensary ---------------------- 2<br>Health Centre -------------------- 3<br>Hospital ------------------------- 4<br>Other ___________________ 99<br>(specify)

PREGNANCY AND CHILDBIRTH AND HISTORY
Now I will ask some questions about your pregnancy and childbirth history.

75. How many children do you have?	_________ children

76. For each of the children listed above (starting with the youngest), please tell me the following:

a. their date of birth

b. where they were delivered

c. who assisted the mother at delivery

Child	a. Child's date of birth<br>(day / month / year)	b. Place of Delivery<br>Home = 1<br>Dispensary = 2<br>Health Centre = 3<br>Hospital = 4<br>In transit = 5<br>Other = (specify)	c. Who assisted delivery?<br>Doctor = 1<br>Nurse = 2<br>Medical Officer = 3<br>Medical Assistant = 4<br>TBA = 5<br>Family member = 6<br>Other = (specify)
1			
2			
3			
4			
5			
6			
7			
8			
9			

77. You mentioned that you would prefer to deliver at __________ but I noticed that most of your children were delivered ___________. Could you tell us why this might be so?

TO BE ASKED FOR EACH CHILD WHERE THERE IS DISCREPANCY.

SELECT ALL THAT ARE MENTIONED.

DO NOT READ ANSWERS ALOUD, SELECT ALL THAT ARE MENTIONED.

Health facility too far away -----------	1
Preferred option too expensive -------	2
Husband/partner insisted --------------	3
Mother in-law insisted -----------------	4
No transportation ----------------------	5
Other __________________________<br>(specify)	99

78. Did a community health worker ever visit you at home during your most recent pregnancy?	YES …………<br>NO …………..	1<br>2
79. If yes, how many times did a community health worker visit you during your most recent pregnancy?	Once -----------------------<br>Twice ----------------------<br>Three times ---------------<br>Four times ----------------<br>More than four times -----	1<br>2<br>3<br>4<br>5
80. If yes, did the CHW use a cell phone as a tool when they visited you during your most recent pregnancy?	YES …………<br>NO …………..	1<br>2
81. If yes, did the CHW use a photo flipbook when they visited you during your most recent pregnancy?<br>(SHOW PHOTO FLIPBOOK)	YES …………<br>NO …………..	1<br>2
82. Has a community health worker visited you since the birth of your most recent baby?	YES …………<br>NO …………..	1<br>2
83. If yes, how many times did a community health worker visit you?	Once -----------------------<br>Twice ----------------------<br>Three times ---------------<br>Four times ----------------<br>More than four times -----	1<br>2<br>3<br>4<br>5
84. If yes, did the CHW use a cell phone as a tool when they visited you during your most recent pregnancy?	YES …………<br>NO …………..	1<br>2
85. If yes, did the CHW use a photo flipbook when they visited you during your most recent pregnancy?<br>(SHOW PHOTO FLIPBOOK)	YES …………<br>NO …………..	1<br>2

This is the end of the survey. Is there anything you would like to ask me?

Would you be interested in participating in future research activities? **Circle one:** YES / NO

Thank you for your time. We appreciate your help with our research.
