# Supplementary material for: Impact of smartphone-assisted prenatal home visits on women’s use of facility delivery: Results from a cluster-randomized trial in rural Tanzania
Source: PLoS One. 2018 Jun 18;13(6):e0199400. doi: 10.1371/journal.pone.0199400 (PMC6005474; doi:10.1371/journal.pone.0199400)
Supplement: S2 Questionnaire — (PDF) [file pone.0199400.s005.pdf]

SIRI: Kwa matumizi ya utafiti tu

UNIVERSITY OF TORONTO

**Utafiti kwa kina mama katika kaya (wateja wa wahudumu wa afya)**

## TAARIFA ZA USIMAMIZI:

| | |
|---|---|
| Jina la mtafiti msaidizi: | Utambulisho wa kijiji:<br><br>----------------- |
| Sahihi ya mtafiti msaidizi: | |
| Tarehe ya usaili:<br><br>-------- / ------- / ----------<br>(siku / mwezi / mwaka) | Utambulisho wa mshiriki:<br><br>----------------- |

| **TAARIFA ZA KIJAMII :** | | |
|---|---|---|
| Wakati wa utafiti tafadhali uliza kama hujaelewa swali. Kwanza nitakuuliza maswali ya ujumla kuhusu wewe na kaya yako kwa ujumla. | | |
| 1. Umezaliwa tarehe ngapi? | -------/-------/--------<br>siku/mwezi/mwaka | |
| 2. Je, una kiwango gani cha elimu? | Hamna ----------------------------<br>Elimu isiyo rasmi---------<br>Elimu ya msingi 1-4 --------------<br>Elimu ya msingi 1-7 --------------<br>Elimu ya sekondari -----------<br>Elimu ya juu ya sekondari<br>Chuo/Chuo kikuu -------------- | 00<br>1<br>2<br>3<br>4<br>5<br>99 |
| 3. Je,kipato chako kwa mwezi kwa ajili ya familia yako ni kiasi gani? | ________________ TSh | |
| 4. Je, ni nini chanzo kikuu cha maji ya kunywa katika kaya | Maji ya bomba --------<br>Maji salama ya kisima-----------<br>Maji ya mvua------------<br>Maji ya chupa--------------------- | 1<br>2<br>3<br>4 |

| | | |
|---|---|---|
| | Maji yasiyosalama ya kisima ---<br>Maji ya mto,dimbwi au mfereji<br>Sifahamu----------<br>Kingine____________________<br>(taja) | 5<br>6<br>88<br>99 |
| 5. Je, wanakaya wako wanatumia aina gani ya choo? | Choo cha kuflashi ------<br>Choo cha shimo -<br>Ndoo ---------------------------<br>Hakuna choo, unatumia vichaka.. -<br>Nyingine___________________<br>(taja) | 1<br>2<br>3<br>4<br>99 |
| 6. Je, ipi ni dini yako? | Mkristo -------------------------<br>Mwislamu -------------------------<br>Sina dini ---------------------------<br>Nyingine____________________<br>(taja) | 1<br>2<br>88<br>99 |
| 7. Je,ni umbali gani ilipo dispensary au kituo cha afya cha karibu( kwa kilomita)? | ___________ kilomita | |
| 8. Je, ni njia gani kuu ya usafiri unayoitumia kwenda kwenye kituo cha afya? | Kwa miguu--------------------------<br>Basi -------------------------------<br>Taksi ------------------------------<br>Baiskeli ---------------------------<br>Pikipiki -----------------------<br>Mwingine____________________<br>(taja) | 1<br>2<br>3<br>4<br>5<br>99 |
| 9. Je, inachukua muda gani kusafiri mpaka kliniki kwa hii njia? | Masaa______<br>Dakika_______ | |
| 10. Je, una watoto wangapi? | watoto_________ | |

**SEHEMU YA KUJIFUNGULIA NA HISTORIA YA MATATIZO YA KIPINDI CHA KUJIFUNGUA:**

Sasa nitakuuliza baadhi ya maswali kuhusu wakati wa ujauzito na historia ya kujifungua

11. Kwa kila mtoto aliyeorodheshwa hapo juu (anza na wa mwisho) tafadhali niambie yafuatayo:

a. Tarehe zao za kuzaliwa

b. Sehemu walipozaliwa

c. Ni nani aliyemzalisha mama

KWA KILA MTOTO,ANDIKA NAMBA YA UTAMBULISHO HUSIKA KATIKA JEDWALI ULIYOPEWA.

| Mtoto | a. Tarehe ya kuzaliwa mtoto<br>(siku/ mwezi / mwaka) | b. Sehemu alipozaliwa<br>Nyumbani = 1<br>Dispensari = 2<br>Kituo cha afya = 3<br>Hospitali= 4<br>Njiani = 5<br>Kwingine = (taja) | c. Ni nani aliyezalisha?<br>Daktari = 1<br>Nesi = 2<br>Mganga mkuu = 3<br>Mganga mkuu msaidizi =4<br>Mkunga wa jadi = 5<br>Wanafamilia= 6<br>Mwingine = (taja) |
|---|---|---|---|
| 1 | | | |
| 2 | | | |
| 3 | | | |
| 4 | | | |
| 5 | | | |
| 6 | | | |
| 7 | | | |
| 8 | | | |
| 9 | | | |

| | | |
|---|---|---|
| 12. Je, unampendelea nani akuhudumie wakati wakujifungua? | Daktari------------------------------------ | 1 |
| | Nesi ------------------------------------ | 2 |
| | Mganga mkuu ------------------------ | 3 |
| | Mganha mkuu msaidizi ---------------- | 4 |
| | Mkunga--------- | 5 |
| | Mwana familia----------------- | 6 |
| | Mwingine__________(taja) | 99 |
| 13. Je, ni sehemu gani unayoipendelea zaidi kujifungulia? | Nyumbani ---------------------------- | 1 |
| | Dispensari ---------------------- | 2 |
| | Kituo cha afya-------------------- | 3 |
| | Hospitali ------------------------- | 4 |
| | Sehemu nyingine_______________ (taja) | 99 |

KAMA SEHEMU ANAYOPENDELEA KUJIFUNGULIA NI TOFAUTI NA SEHEMU ANAYOJIFUNGULIA WATOTO WAKE WOTE ULIZA SWALI #14. KAMA SEHEMU ANAYOJIFUNGULIA NA SEHEMU ANAYOPENDELEA KUJIFUNGULIA NI SAWA RUKA HADI SWALI #15.

| | | |
|---|---|---|
| 14. Umetaja kuwa unapendelea kujifungua katika __________ lakini nimegundua kuwa karibia watoto wako wote umejifungulia___________. Unaweza kutuambia kwa nini hii iko hivi | Kituo cha afya Kiko mbali sana ------ | 1 |
| | Sehemu ninapopapendelea ni ghali -- | 2 |
| | Mume/mwenza alisisitiza ------------ | 3 |
| | Mama mkwe alisisitiza ---------------- | 4 |
| | Hakuna usafiri ---------------------- | 5 |
| | Mengineyo____________________ | 99 |

| | | |
|---|---|---|
| | (taja) | |
| 15. Je, mhudumu wa afya ameshawahi kukutembelea nyumbani kwako katika kipindi cha ujauzito wako wa hivi karibuni? | NDIO………… <br> HAPANA…………… | 1 <br> 2 |
| 16. Kama ndio,ni mara ngapi mhudumu wa afya alikutembelea? | Mara moja ----------------------- <br> Mara mbili---------------------- <br> Mara tatu --------------- <br> Mara nne---------------- <br> Zaidi ya mara nne ----- | 1 <br> 2 <br> 3 <br> 4 <br> 5 |
| 17. Je, mhudumu wa afya alikutembelea ulipo jifungua mtoto wako wa wa hivi karibuni? | NDIO ………… <br> HAPANA …………… | 1 <br> 2 |
| 18. Kama ndio,ni mara ngapi mhudumu wa fay alikutambele? | Mara moja ----------------------- <br> Mara mbili ---------------------- <br> Mra tatu --------------- <br> Mara nne---------------- <br> Zaidi ya mara nne ----- | 1 <br> 2 <br> 3 <br> 4 <br> 5 |

**HUDUMA ZA AFYA ZINAZOTOLEWA NA WAHUDUMU WA AFYA**

Ningependa kujua mawazo yako kuhusu huduma zinazotolewa na wahudumu wa afya,.Nitakusomea baadhi ya sentensi.Nikisha kusomea sentensi tafadhali niambie kama “unakubaliana kabisa’, “unakubaliana”,”haukubalianai”,”haukubaliani kabisa”

| | Ninakubaliana kabisa | Ninakubaliana | Sikubaliani | Sikubaliani kabisa | Sina uhakika | |
|---|---|---|---|---|---|---|
| 19. Wahudumu wa afya wanajua kiundani kuhusu afya za wakina mama | 3 | 2 | 1 | 0 | 99 | |
| 20. Wahudumu wa afya wanajua kiundani kuhusu afya ya mtoto | 3 | 2 | 1 | 0 | 99 | |
| 21. Wahudumu wa afya hawana uwezo mkubwa wa kutambua magonjwa ya mtoto. | 0 | 1 | 2 | 3 | 99 | |
| 22. Ninauamini ushauri wa kiafya unaotolewa na wahudumu wa afya | 3 | 2 | 1 | 0 | 99 | |
| 23. Wahudumu wa afya wana uwezo mkubwa wa ufuatiliaji wa wateja wao kila wakati. | 3 | 2 | 1 | 0 | 99 | |
| 24. Wahudumu wa afya wana ujuzi wa kutosha. | 3 | 2 | 1 | 0 | 99 | |
| 25. Wahudumu wa afya hawatoi ufumbuzi mzuri wa matatizo ya kiafya katika familia yangu | 0 | 1 | 2 | 3 | 99 | |
| 26. Wahudumu wa afya wana vifaa stahiki katika kuwahudumia wakina mama na watoto wadogo. | 0 | 1 | 2 | 3 | 99 | |

| **MAONI KUHUSU MTU BINAFSI KUKATIKA HUDUMA** | | | | | | |
|---|---|---|---|---|---|---|
| Majibu yako katika sentensi zifuatavyo yataniwezesha kujifunza zaidi juu ya uhusiano wako na wahudumu wa afya katika eneo lako. Kwa mara nyingine tafadhali niambie kama "unakubaliana kabisa", "unakubaliana", "haukubaliani", or "haukubaliani kabisa". | | | | | | |
| KUJALI | **Unakubaliana kabisa** | **Unakubaliana** | **Haukubaliani** | **Haukubaliani kabisa** | **Hauna uhakika** | |
| 27. Wahudumu wa afya wanakujali wewe pamoja na watoto wako | 3 | 2 | 1 | 0 | 99 | |
| 28. Wahudumu wa afya hawakujali wewe pamoja na mtoto wako | 0 | 1 | 2 | 3 | 99 | |
| 29. Wahudumu wa afya hawana muda wa kutosha wa kukaa na kimama na watoto wanaowahudumia | 0 | 1 | 2 | 3 | 99 | |
| 30. Ni rahisi kuongea na mhudumu wa afya | 3 | 2 | 1 | 0 | 99 | |
| 31. Ninaamini wahudumu wa afya ni waaminifu | 3 | 2 | 1 | 0 | 99 | |
| 32. Kwa ujumla, ninaridhika na huduma zinazotolewa katika familia yangu na wahudumu wa afya | 3 | 2 | 1 | 0 | 99 | |

| Sasa nitakuuliza baadhi ya maswali kuhusu kituo cha afya kilicho karibu yako na kuhusu wahudumu wanaofanya kazi hapo.Kwa mara nyingine baada ya kusoma sentensi,tafadhali niambie kama "unakubaliana kabisa", "unakubaliana", "haukubaliani", or "haukubaliani kabisa". | | | | | | |
|---|---|---|---|---|---|---|
| | **Unakubaliana kabisa** | **Unakubaliana** | **Haukubaliani** | **Haukubaliani kabisa** | **Hauna uhakika** | |
| 33. Wafanyakazi wa kituo cha afya wananijali mimi na watoto wangu. | 3 | 2 | 1 | 0 | 99 | |
| 34. Wafanyakazi wa kituo cha afya hawanijali mimi na watoto wangu. | 0 | 1 | 2 | 3 | 99 | |
| 35. Wafanya kazi wa kituo cha afya hawana mda wa kutosha kukaa na wamama na watoto wanaowahudumia | 0 | 1 | 2 | 3 | 99 | |
| 36. Nirahisi kuongea na wafanyakazi wa kituo cha afya | 3 | 2 | 1 | 0 | 99 | |
| 37. Ninaamini ushauri wa kiafya unaotolewa na mfanyakazi wa kituo cha afya. | 3 | 2 | 1 | 0 | 99 | |
| 38. Wafanyakazi wa kituo cha afya wanaufahamu kuhusu afya ya mama na mtoto. | 3 | 2 | 1 | 0 | 99 | |
| 39. Wafanyakazi wa vituo vya afya ni wakatili kwa wagonjwa. | 0 | 1 | 2 | 3 | 99 | |

| | | | | | | |
|---|---|---|---|---|---|---|
| 40. Kituo cha afya ni kisafi na kinatunzwa vizuri daima | 3 | 2 | 1 | 0 | 99 | |
| 41. Kituo cha afya kina vifaa stahiki . | 3 | 2 | 1 | 0 | 99 | |
| 42. Mara nyingi kituo cha afya kinapungukiwa na dawa na vifaa vingine. | 0 | 1 | 2 | 3 | 99 | |
| 43. Kituo cha afya kina wafanya kazi wa kutosha | 3 | 2 | 1 | 0 | 99 | |
| 44. Foleni kwenye kituo cha afya ni ndefu sana. | 0 | 1 | 2 | 3 | 99 | |
| 45. Kwa ujumla,ninaridhika na huduma inayotolewa kwa familia yangu na wafanyakazi wa kituo cha afya. | 3 | 2 | 1 | 0 | 99 | |

**UJAUZITO WA HIVI KARIBUNI NA KUJIFUNGUA**

Maswali yafuatayo yanahusu ujauzito wako wa hivi karibuni na kujifungua
*Muhimu kwa mtafiti:
Pale itakapofaa,tafadhali thibitisha taarifa ifuatayo kwa kuuliza ili kuona kadi ya kliniki ya mshiriki kama ipo

| | | |
|---|---|---|
| 46. Je, ulijifungua mtoto wako wa hivi karibuni katika kituo cha afya? | NDIO ……..…… | 1 |
| | HAPANA …………….. | 2 |
| 47. Kama ndio,hiki kituo kilikuwa karibu na wewe? | NDIO……..…… | 1 |
| | HAPANA…………….. | 2 |
| 48. Kama hukujifungua katika kituo cha afya,tafadhali tuambie ulipojifungulia . | Nyumbani …......... | 1 |
| | Njiani kuelekea kituoni …… | 2 |
| | Pengine ________________ | 99 |
| 49. Kama hukujifungulia katika kituo cha afya,je unaweza kuniambia kwa nini? | Ninapendelea kujifungulia nyumbani------------ | 1 |
| | Kituo cha afya kiko mbali sana ------ | 2 |
| | Huduma za afya ni ghali --------- | 3 |
| | Mume/mwenza alisisitiza ------------ | 4 |
| | Mama mkwe alisisitiza--------------- | 5 |
| | Hakuna usafiri --------------------- | 99 |
| | Kingine______________________ (taja) | |
| 50. Je, ulipokea huduma za kliniki baada ya kujifungua katika kituo cha afya katika kipindi cha siku 2-3 baada ya kujifungua? (*angalia kadi ya kliniki ) | NDIO……..…… | 1 |
| | HAPANA…………….. | 2 |
| 51. Je, ulihudhuria kliniki mara ngapi katika kipindi cha ujauzito katika kituo cha afya wakati wa kipindi cha mimba yako ya hivi karibuni? (*angalia kadi ya kliniki ) | Moja ……………… | 1 |
| | Mbili ..…………… | 2 |
| | Tatu ……………. | 3 |
| | Nne ………..…… | 4 |

| | | |
|---|---|---|
| | Hakuna ……………..<br>Zaidi ya mara nne……… | 0<br>99 |
| 52. Je,ulikuwa na mimba ya miezi mingapi mara ya kwanza wakati ulipoenda kliniki ? (*angalia kadi ya kliniki) | Idadi ya miezi | _____ |
| 53. Je, ulipokea au ulinunua vidonge ili kuongeza damu wakati ulipokuwa na ujauzito wa huyu mtoto? (madini ya chuma) (onyesha kidonge) | NDIO ……..…..<br>HAPANA ……..…….. | 1<br>2 |
| 54. Sina nia ya kujua matokeo yako, lakini ulipima ukimwi katika kipindi hichi cha ujauzito? | NDIO ……..…..<br>HAPANA ……..…….. | 1<br>2 |
| 55. Sina nia ya kujua matokeo yako,lakini mme/mwenza wako alipima ukimwi wakati wa hiki kipindi cha ujauzito? | NDIO……..…..<br>HAPANA……..…….. | 1<br>2 |
| 56. Je, ulitumia vidonge vya minyoo wakati wa ujauzito? | NDIO ……..…..<br>HAPANA ……..…….. | 1<br>2 |
| 57. Je,unachandarua chenyedawa ya kuulia mmbu nyumbani? | NDIO……..…..<br>HAPANA ……..…….. | 1<br>2 |
| 58. Kama ndio, ni nani anayekitumia chandarua chenye dawa? | Mume wangu tu -------------------------<br>Mimi tu ----------------------------<br>Mume wangu na mimi tu -----------<br>Mtoto/watoto tu -----------------------<br>Mume wangu, mimi na watoto | 1<br>2<br>3<br>4<br>5 |
| 59. Je ni mara ngapi umetumia neti katika kipindi chako cha mwisho cha ujauzito? | Kila usiku ---------------------------<br>Karibia kila usiku ------------------------<br>Baadhi ya usiku --------------------------<br>Sijawahi/sio kila mara -------------------- | 1<br>2<br>3<br>0 |
| 60. Je, ulitumia net jana usiku? | NDIO……..…..<br>HAPANA……..…….. | 1<br>2 |
| 61. Je, ulitumia dozi ngapi za malaria kipindi cha ujauzito ? (*angalia kadi ) | MOJA………….<br>MBILI…………<br>HAKUNA ………. | 1<br>2<br>0 |
| 62. Je, ulipokea chanjo ngapi za tetanus kipindi cha ujauzito? (* angalia kadi) | MOJA………….<br>MBILI…………<br>HAKUNA ………. | 1<br>2<br>0 |
| 63. Je,ni mara ngapi mhudumu wa afya alikutembelea katika kipindi cha mwisho cha ujauzito wako? | tembelea-------------------- | |
| 64. Ulisha wahi kupewa rufaa na mhudumu wa afya kwenda kituo | NDIO……..….. | 1 |

| | | |
|---|---|---|
| cha afya katika kipindi hicho cha ujauzito? | HAPANA …………… | 2 |
| 65. Kama ulipewa rufaa na mhudumu wa afya kijijini, ulienda kliniki? | NDIO …………<br>HAPANA……………<br>Kama sio, kwa nini?<br>_______________________________ | 1<br>2 |
| 66. Mme/mwenza wako alikusindikiza katika mwito wowote wa kupatiwa huduma za kipindi cha ujauzito? | NDIO…………<br>HAPANA……………<br>Kama ndio mara ngapi? ______ | 1<br>2 |
| 67. Je mme/mwenza wako alikusindikiza wakati wa kipindi cha mwisho cha kujifungua? | NDIO…………<br>HAPANA…………… | 1<br>2 |
| 68. Je, ulikuwa na mpango binafsi wa kujifungua kabla ya kujifungua? | NDIOYO…………<br>HAPANA…………… | 1<br>2 |
| 69. Kama ndio,ni nani anayekusaidia kuandaa mpango binafsi wa kujifungua? | Wafanyakazi wa huduma za afya----<br>Mhudumu wa afya kijijini<br>Mkunga wa jadi --------------------------------<br>Wanafamilia --------------------<br>Wengine: ______________________ (taja) | 1<br>2<br>3<br>4<br>99 |
| 70. Je,ulipanga ujifungulie wapi? | Nyumbani ----------------------------<br>Dispensari-------------------------<br>Hospitali ----------------------------<br>Nyumbani kwa ndugu ---------------<br>kwingine: _____________________ (taja) | 1<br>2<br>3<br>4<br>99 |
| 71. Huyu mtoto alipewa matone ya polio alipozaliwa? (*angalia kadi ya mtoto) | NDIO…………<br>HAPANA…………… | 1<br>2 |
| 72. Je, umeshawahi kumnyonyesha huyu mtoto? | NDIO…………<br>HAPANA……………<br>**kama sio ruka nenda swali la 51* | 1<br>2 |
| 73. Je, ulimnyonyesha kwa mda gani mtoto huyu baada ya kujifungua? | masaa<br>siku<br>*kama ni chini ya lisaa limoja rekodi masaa"00" . Kama ni chini ya* | _____<br>_____ |

| | | |
|---|---|---|
| | *masaa 24 rekodi masaa. Vinginevyo rekodi siku .* | |
| 74. Kulikuwa na kimiminika au chakula alichopewa mtoto wiki ya kwanza baada ya kujifungua? | NDIO………… | 1 |
| | HAPANA…………… | 2 |
| | Kama ndio,tafadhali orodhesha | |
| 75. Je,unaendelea kumnyonyesha huyu motto? | NDIO………… | 1 |
| | HAPANA …………… | 2 |
| 76. Sasa ningependa kukuuliza kuhusu vimiminika au vyakula( taja jina la motto) alichopata jana mchana au usiku. Je, mtoto(taja jina la mtoto) alikunywa au alikula?: | a. Maziwa ya mama ........................... | Y N |
| | b. Maji matupu................................... | Y N |
| | c. Lishe ya mtoto ............................... | Y N |
| ** Kwa kila jibu sahihi zungushia Y au N* | d. Lishe yoyote iliyoandaliwa ya kibiashara ya wachanga au watoto wadogo(kwa mfano; taja jina la hiyo kampuni) …. | Y N |
| | e. (Kingine) chochote,uji au chakula cha kimiminika….. ........................... | Y N |
| 77. Je, mtoto alipewa chanjo ya kifua kikuu katika kipindi cha wiki ya kwanza baada ya kuzaliwa? (*angalia kadi) | NDIO………… | 1 |
| | HAPANA…………… | 2 |
| 78. Tungependa kujua uelewa wako juu ya dalili za hatari kipindi cha ujauzito.Je unaweza kutaja dalili za hatari ambazo unazifahamu? | | |
| ***Orodhesha kila kilichotajwa.Kama hakuna andika "00"→** | | |
| 79. Tungependa kujua kuhusu uelewa wako juu ya dalili za hatari ambazo unazijua kipindi cha kujifungua. Je unaweza kutaja dalili zozote za hatari unazozijua? | | |

| | |
|---|---|
| ***Ziorodheshe dalili zote zinazotajwa.Kama hamna andika "00"→*** | ______<br>______<br>______<br>______ |
| ***80.*** Katika kipindi cha nyuma,ulishakutana na ugumu au matatizo wakati unajifungua?( kwa mtoto wako yeyote?)<br>*Kama hapana,ruka nenda swali linalofuata | NDIO---------------- 1<br>HAPANA ------------------ 2<br>SINAUHAKIKA----------- 3 |
| 81. Kama ndio, ulikutana na matatizo gani? | **Orodhesha matatizo yote yaliyotajwa** :<br>______<br>______<br>______<br>______ |

Huu ni mwisho wa utafiti.Je kuna kitu chochote ungependa kuniuliza?
Ungependa kushiriki tena katika kazi za utafiti ujao?Zungushia moja**:** NDIO /HAPANA

Asante kwa mda wako.Tunathamini msaada wako katika utafiti wetu
