# Supplementary material for: Impact of smartphone-assisted prenatal home visits on women’s use of facility delivery: Results from a cluster-randomized trial in rural Tanzania
Source: PLoS One. 2018 Jun 18;13(6):e0199400. doi: 10.1371/journal.pone.0199400 (PMC6005474; doi:10.1371/journal.pone.0199400)
Supplement: S1 Supporting Information — (PDF) [file pone.0199400.s006.pdf]

Professor Daniel Sellen and Dr. Kristy Hackett

Dalla Lana School of Public Health
University of Toronto

March 10, 2017

Dear Professor Sellen and Dr. Hackett,

I am writing to confirm the determination I made four years ago with respect to whether your project, entitled "Evaluation of an mHealth intervention to improve women's access to maternal health in rural Tanzania" fits the criteria for clinical trial registration.

As stated back then, the International Committee of Medical Journal Editors (ICJME) defined a clinical trial as "any research that prospectively assigns human participants or groups to one or more health-related interventions to evaluate effects on health outcomes".

Based on the submitted and approved ethics protocol submission, it is clear that the study that was conducted did not meet the definition of clinical trial for several reasons. These include:

1. The primary participant group for this study was community health workers (CHWs) who were using the intervention, the mHealth app, or standard tools (cell phone, paper) in an administrative capacity.
2. The evaluation was on the utility and helpfulness of this app to their work, data collected primarily through qualitative means including surveys, interviews and focus groups.
3. The secondary participant group was patients whose CHWs may have used the mHealth app, cell phone, or paper to conduct their clinical work. There was no direct intervention for this group, instead, they were asked to evaluate their experiences with the CHWs and to provide details on delivery location of their babies.

I remain confident that this determination was correct and no registration of your study is required through clinicaltrials.gov.

Please let me know if you have any questions or concerns.

Sincerely yours,

RZand

Rachel Zand, Ph.D.
Director, Human Research Ethics
